# Myocarditis occurrence with cancer immunotherapy across indications in clinical trial and post-marketing data

**DOI:** 10.1038/s41598-021-96467-5

**Published:** 2021-08-30

**Authors:** Tigran Makunts, Ila M. Saunders, Isaac V. Cohen, Mengxing Li, Talar Moumedjian, Masara A. Issa, Keith Burkhart, Peter Lee, Sandip Pravin Patel, Ruben Abagyan

**Affiliations:** 1 Oak Ridge Institute of Science and Education Fellowship at Office of Clinical Pharmacology, United States Food and Drug Administration, Silver Spring, MA USA; 2 Skaggs School of Pharmacy and Pharmaceutical Sciences, University of California San Diego, La Jolla, CA USA; 3 Clinical Pharmacology and Therapeutics (CPT) Postdoctoral Training Program, University of California San Francisco, San Francisco, CA USA; 4 Center for Drug Evaluation and Research, US Food and Drug Administration, Silver Spring, MA USA; 5 Center for Personalized Cancer Therapy, Division of Hematology and Medical Oncology, UCSD Moores Cancer Center, La Jolla, CA USA

**Keywords:** Cardiomyopathies, Oncology, Cancer, Cancer therapy, Cancer immunotherapy, Adverse effects, Immunotherapy, Cancer, Cancer therapy, Cancer immunotherapy

## Abstract

Antibodies targeting the PD-1, PD-L1, and CTLA-4 immune checkpoint axis have been used in a variety of tumor types. They achieve anti-tumor activity through activating the patient’s own immune system to target immune response evading cancer cells. However, this unique mechanism of action may cause immune-related adverse events, irAEs. One of these irAEs is myocarditis which is associated with an alarming mortality rate. In this study we presented clinical cases of myocarditis from safety trial datasets submitted to the U.S. Food and Drug Administration, FDA. Additionally, we analyzed over fourteen million FDA Adverse Event Reporting System, FAERS, submissions. The statistical analysis of the FAERS data provided evidence of significantly increased reporting of myocarditis in patients administered immune checkpoint inhibitors alone, in combination with another immune checkpoint inhibitor, the kinase inhibitor axitinib, or chemotherapy, for all cancer types, when compared to patients administered chemotherapy. All combination therapies led to further increased reporting odds ratios of myocarditis. We further analyzed the occurrence of myocarditis by stratifying the reports into sub-cohorts based on specific cancer types and treatment/control groups in major cancer immunotherapy efficacy trials and confirmed the observed trend for each cohort.

## Introduction

The field of cancer immunotherapy has continuously gained appreciation with the success of various targeted immune checkpoint inhibitors (ICIs). Malignant cancer cells have the capacity to evade the immune system by suppressing the activation of T-cells^1^. This concept led to the discovery of new strategies to block the immune checkpoint breaks and re-activate the immune response^1^. The first immunotherapy antibody approval in 2011, ipilimumab^2^, targeted the cytotoxic T-lymphocyte–associated antigen 4 (CTLA-4)^2^. Antibodies targeting the programmed cell death protein 1 (PD-1) receptors, pembrolizumab and nivolumab were approved in 2014, and cemiplimab in 2018. Combination therapies, such as pembrolizumab plus axitinib and nivolumab plus ipilimumab, also received approvals for various indications. Monoclonal antibodies targeting the PD-1 ligand (PD-L1) atezolizumab (2016), durvalumab (2017), and avelumab (2017)^1^ were also recently approved.

The use of the checkpoint inhibitors has been linked to serious immune-related adverse events (irAEs)^3^ including rare but potentially fatal cardiac toxicity such as myocarditis^4^.

Myocarditis was observed in < 1% of patients receiving ICI therapy, with cardiac rhythm disturbances as the initial presentation^3^. A randomized, double-blind, placebo-controlled trial evaluating the safety of ipilimumab (CA184-029), a CTLA-4 inhibitor, in adjuvant treatment of melanoma found a severe to fatal myocarditis incidence of 0.2%^5^. Myocarditis as a fatal adverse reaction was also reported for ipilimumab in combination with PD-1 blocker nivolumab as a first-line treatment for non-small-cell lung cancer (CHECKMATE-227)^5^. A study evaluating PD-1 blocker pembrolizumab for the treatment of classical Hodgkin Lymphoma (KEYNOTE-087) reported a myocarditis incidence of 0.5%^6^. The prescribing information of cemiplimab listed autoimmune myocarditis as one of the adverse reactions occurring in less than 5% of patients^7^. Similarly, in the prescribing information of PD-L1 blockers atezolizumab, durvalumab and avelumab, myocarditis is listed as a clinically significant irAE occurring in < 1% of the patients^8–10^. In a phase-III study evaluating avelumab in combination with axitinib for the treatment of advanced renal cell carcinoma (JAVELIN Renal 101), death associated with myocarditis, and necrotizing pancreatitis occurred in 3 patients in the avelumab-plus-axitinib cohort (0.7%)^11^. Although only a few cases of myocarditis incidence were described in clinical trials (Table 1), the severity and possible fatality of the disease remained under-characterized, prompting further investigation.

**Table 1:** Summary of Myocarditis occurrence in clinical trials for immune checkpoint inhibitors.

Drug	Initial US approval	Labeled indications	Efficacy trial	Drug	Control	Myocarditis incidence
Ipilimumab/YERVOY, CTLA-4	2011	Unresectable or Metastatic Melanoma, Adjuvant Treatment of Melanoma^5^ In combination with Nivolumab: Advanced Renal Cell Carcinoma (RCC), Microsatellite Instability-High (MSI-H) or Mismatch Repair Deficient (dMMR) Metastatic Colorectal Cancer, Hepatocellular Carcinoma Metastatic Non-Small Cell Lung Cancer (NSCLC)^5^	Melanoma: MDX010-20^12^ Metastatic NSCLC: CHECKMATE-227^13^	Ipilimumab monotherapy, Ipilimumab in combination with a melanoma peptide vaccine Nivolumab, or Nivolumab + Ipilimumab, or Nivolumab + Platinum-doublet Chemotherapy	Melanoma Vaccine Monotherapy Platinum Doublet Chemotherapy	From prescribing Information in Adjuvant treatment of Melanoma: severe to fatal, 0.2% (CA184-029)^a^ In first-line Treatment of Metastatic NSCLC: In Combination with Nivolumab (CHECKMATE-227)^b^
Pembrolizumab/KEYTRUDA, PD-1	2014	Melanoma, Non-Small Cell Lung Cancer (NSCLC), Head and Neck Squamous Cell Cancer, Classical Hodgkin Lymphoma (cHL), Primary Mediastinal Large B-Cell Lymphoma, Urothelial Carcinoma, Microsatellite Instability-High Cancer, Gastric Cancer, Cervical Cancer, Hepatocellular Carcinoma, Merkel Cell Carcinoma^6^. In combination with Axitinib: first-line treatment against advanced/metastatic Renal Cell Carcinoma (mRCC) (https://www.fda.gov/drugs/drug-approvals-and-databases/fda-approves-pembrolizumab-plus-axitinib-advanced-renal-cell-carcinoma)	Melanoma: KEYNOTE-006^14^ Classical Hodgkin Lymphoma: Phase II KEYNOTE-087^15^ mRCC: KEYNOTE-426^16^ NSCLC: KEYNOTE 189^17^ NSCLC: KEYNOTE—407^18^	Pembrolizumab Pembrolizumab Pembrolizumab + axitinib Pembrolizumab + pemetrexed + platinum-based chemotherapy Pembrolizumab + carboplatin + paclitaxel or nab-paclitaxel	Ipilimumab Single arm, Non-randomized Sunitinib Placebo + pemetrexed + platinum-based chemotherapy Placebo + carboplatin + paclitaxel or nab-paclitaxel	In Classical Hodgkin Lymphoma: 0.5% (KEYNOTE-087)^c^ In mRCC: Of the 11 patients who died from adverse events in the combination group, 1 died from myocarditis
Nivolumab/OPDIVO, PD-1	2014	Unresectable or Metastatic Melanoma, Adjuvant Treatment of Melanoma, Metastatic NSCLC, Small Cell Lung Cancer, Advanced RCC, cHL, Squamous Cell Carcinoma of the Head and Neck, Urothelial Carcinoma, Microsatellite Instability-High or Mismatch Repair Deficient Metastatic Colorectal Cancer, Hepatocellular Carcinoma, Esophageal Squamous Cell Carcinoma (ESCC)^19^	Advanced Melanoma: CHECKMATE-037^20^ Metastatic NSCLC in combination with Ipilimumab: CHECKMATE-227^13^	Nivolumab Nivolumab, or Nivolumab + Ipilimumab, or Nivolumab + Platinum-doublet Chemotherapy	Either Dacarbazine or Carboplatin and Paclitaxel Platinum Doublet Chemotherapy	In metastatic NSCLC: (CHECKMATE-227)^e^
Cemiplimab/LIBTAYO, PD-1	2018	Metastatic Cutaneous Squamous Cell Carcinoma (CSCC) or locally advanced CSCC^7^	Study 1423 and 1540^21^	Cemiplimab, Cemiplimab + anti-cancer therapy (radiotherapy, cyclophosphamide, docetaxel, carboplatin, GM-CSF, paclitaxel, pemetrexed)		From prescribing information^f^
Atezolizumab/TECENTRIQ, PD-L1	2016	Urothelial Carcinoma, NSCLC, Locally Advanced or Metastatic Triple-Negative Breast Cancer, Small Cell Lung Cancer (SCLC), Hepatocellular Carcinoma^8^	Urothelial Carcinoma: IMvigor210^22^ Non-squamous NSCLC: Impower150^23^	Atezolizumab Atezolizumab in Combination with Carboplatin + Paclitaxel with or without Bevacizumab	Carboplatin + paclitaxel + bevacizumab	From prescribing information^g^
Durvalumab/IMFINZI, PD-L1	2017	Urothelial Carcinoma, NSCLC, SCLC^9^	Urothelial Carcinoma: Study 1108^24^ NSCLC: PACIFIC^25^ SCLC: CASPIAN^26^	Durvalumab Durvalumab Durvalumab ± tremelimumab with platinum-based chemotherapy (carboplatin or cisplatin + etoposide)	Placebo Platinum-based chemotherapy	From prescribing information^h^
Avelumab/BAVENCIO, PD-L1	2017	Metastatic Merkel Cell Carcinoma, Locally Advanced or Metastatic Urothelial Carcinoma^10^In combination with Axitinib: first-line for advanced RCC^10^	Metastatic Merkel Cell Carcinoma: JAVELIN Merkel 200^27^ Urothelial Carcinoma: JAVELIN Solid Tumor^28^ Advanced RCC in combination with Axitinib: JAVELIN Renal 101^11^	Avelumab Avelumab Avelumab + axitinib	Sunitinib	In Advanced RCC in combination with axitinib (JAVELIN Renal 101): 0.2%^i^ From prescribing information^j^

*RCC* renal cell carcinoma, *mRCC* metastatic renal cell carcinoma, *NSCLC* non-small cell lung cancer, *SCLC* small cell lung cancer, *CSCC* cutaneous squamous cell carcinoma.

^a^In CA184-029, the following clinically significant irAEs were seen in less than 1% of YERVOY-treated patients unless specified: cytopenias, eosinophilia (2.1%), pancreatitis (1.3%), meningitis, pneumonitis, sarcoidosis, pericarditis, uveitis, and fatal myocarditis [see Adverse Reactions (6.1)]^5^.

^b^Fatal adverse reactions occurred in 1.7% of patients; these included events of pneumonitis (4 patients), myocarditis, acute kidney injury, shock, hyperglycemia, multi- system organ failure, and renal failure^5^.

^c^Other clinically important adverse reactions that occurred in less than 10% of patients on KEYNOTE-087 included infusion reactions (9%), hyperthyroidism (3%), pneumonitis (3%), uveitis and myositis (1% each), and myelitis and myocarditis (0.5% each)^6^.

^d^Of the 11 patients (2.6%) in the pembrolizumab–axitinib group who died from adverse events, 4 (0.9%) died from treatment-related adverse events (from myasthenia gravis, myocarditis, necrotizing fasciitis, and pneumonitis, in 1 patient each)^16^.

^e^Fatal adverse reactions occurred in 1.7% of patients; these included events of pneumonitis (4 patients), myocarditis, acute kidney injury, shock, hyperglycemia, multi-system organ failure, and renal failure^5^.

^f^LIBTAYO was permanently discontinued due to adverse reactions in 5% of patients; adverse reactions resulting in permanent discontinuation were pneumonitis, autoimmune myocarditis, hepatitis, aseptic meningitis, complex regional pain syndrome, cough, and muscular weakness^7^.

^g^The following clinically significant irAEs occurred at an incidence of < 1% in 2616 patients who received TECENTRIQ as a single-agent and in 2421 patients who received TECENTRIQ in combination with platinum-based chemotherapy or were reported in other products in this class^8^.

^h^The following clinically significant irAEs occurred at an incidence of less than 1% each in 1889 patients who received IMFINZI: aseptic meningitis, hemolytic anemia, immune thrombocytopenic purpura, myocarditis, myositis, and ocular inflammatory toxicity, including uveitis and keratitis^9^.

^i^Death due to toxicity of trial treatment that occurred in 3 patients in the avelumab-plus-axitinib group (0.7%) was attributed to sudden death, myocarditis, and necrotizing pancreatitis^11^.

^j^The following irAEs occurred at an incidence of less than 1% of patients who received BAVENCIO as a single agent or in 489 patients who received BAVENCIO in combination with axitinib: immune-mediated myocarditis including fatal cases, pancreatitis including fatal cases, immune-mediated myositis, psoriasis, arthritis, exfoliative dermatitis, erythema multiforme, pemphigoid, hypopituitarism, uveitis, Guillain-Barré syndrome, and systemic inflammatory response^10^.

The term “myocarditis” describes a wide range of pathologies that clinically manifest as an inflammatory condition of the heart muscle occurring alone or as part of a multiorgan immune-mediated disorder or reaction to exogenous or endogenous substances^29^. The altered immune-mediated reactions are the cause of structural and functional abnormalities in the myocardium which are responsible for a variety of injuries to the heart such as contractile impairment, chamber stiffening and conduction system irregularities. Most commonly, myocarditis is categorized according to the major histopathologic pattern which is descriptive of the different etiologies of the disease. The heterogeneity and wide spectrum of clinical manifestations present challenges to the proper diagnosis of the disease and treatment decisions^30^. ICI use has been reported as an etiologic factor contributing to rare, but severe cases of myocarditis, according to World Health Organization (WHO) database^31^. Moslehi et al. describe 101 cases of severe myocarditis following immune checkpoint inhibitor treatment across various cancer types, with a higher frequency reported for the PD-1 and PDL-1/CTLA-4 combination with respect to monotherapy^31^. In a large systematic review and meta-analysis of the ICI-associated irAEs, myocarditis was found to have the highest fatality rate compared to other irAEs (52 of 131 reported cases)^32^. Another retrospective study investigating a total of 36,848 toxicities of immunotherapies reported through FAERS in 2017–2018 described a 6.3% rate of cardiovascular toxicities including myocarditis. The fatality rate of the myocarditis cases was determined to be 50%^33^.

Overall, ipilimumab, nivolumab, pembrolizumab and combinations of PD-1/PDL-1 and CTLA-4 inhibitors are associated with myocarditis at higher rate^34^. In an analysis of post-marketing surveillance data, avelumab had a higher association with myocarditis out of six monotherapy ICI post-marketing reports^35^. In a 2016 study, Johnson et al. describe two case reports of lethal myocarditis accompanied with myositis in patients administered ipilimumab–nivolumab^36^. In another case report from 2019, Saibil et al., described an example of fatal fulminant myocarditis combined with myositis following administration of a single dose of ipilimumab–nivolumab in a patient with stage IV melanoma. The patient presented at day 16 with a history of increasing fatigue, weakness, and dyspnea ultimately progressing to respiratory failure. The histologic patterns observed were myocyte calcification and myocyte lysis with associated inflammatory response^37^.

The manifestations of ICI-associated myocarditis seem to differ compared to general myocarditis suggesting the presence of distinct risk factors^38^. Analysis of the data from a multicenter registry of 8 sites reported half of the patients with myocarditis to have experienced major adverse cardiac events which include cardiogenic shock, cardiac arrest, complete heart block and cardiovascular death. Troponin elevation and abnormal ECG were common findings in most of the clinical cases. In addition, diabetes, sleep apnea and a higher body mass index were among the patient characteristics associated with a higher incidence of myocarditis compared to controls^39^. Also, in their analysis of adverse event reports, Zamami et al., found a significantly higher risk of myocarditis in female patients and patients 75 years and older in the context of ICI compared to general myocarditis independent of ICI treatment^38^. A case series identified 5 out of 8 patients to have pre-existing cardiac pathologies suggesting that myocarditis might manifest as a possible worsening of general cardiac conditions^40^. Co-occurrence of other immune-related adverse events such as myositis, myasthenia gravis, thyroiditis, uveitis, colitis, and hepatitis were also described in the literature^32,40^. As far as treatment, Mahmood et al., observed that the course of the disease was overall responsive to higher doses of steroids which were administered in 89% of cases. However, myocarditis fatalities still occurred despite steroid therapy^39^. Although rare, myocarditis poses a high risk to the patients due to high risk of mortality and warrants further investigation into this irAE.

In this study we used two data sources for a thorough evaluation for the myocarditis cases in clinical trial and postmarketing safety reports submitted to the FDA, including disease progression, preceding irAEs, demographic parameters, CTCAE^41^ toxicity grading, and concomitant oncology medications. The first source, Integrated Summaries of Safety^42^ (ISS), includes the safety information from clinical trials, submitted to the United States Food and Drug Administration (FDA) with New Molecular Entity (NME) and non-NME submissions.

As the second source, we analyzed the FDA FAERS/AERS database for myocarditis reports in patients taking ICIs as monotherapy, ICI-ICI combinations, and ICI in combination with axitinib compared to chemotherapy reports irrespective of indication. We further compared and contrasted myocarditis reported frequencies replicating the study cohorts in the efficacy clinical trials and matching adverse event by indication, treatment, and control groups.

## Methods

### Case studies from ISS data sets

Center for Drug Evaluation and Research electronic NME and non-NME submissions, including ISS and Clinical Safety Summaries (CSS) are maintained in the Electronic Document Room^43^. The ISS component of the Biologic License Applications (BLAs) of interest were mined for Analysis Datasets of Adverse Events (ADAE) for the submissions of ipilimumab, pembrolizumab, nivolumab, cemiplimab, avelumab, atezolizumab, and durvalumab. A total of 24,567 reports, ICI subjects (N = 20,062) and controls (4505), were collected from BLA submissions. The ADAE sets were scanned for myocarditis, immune mediated myocarditis, and autoimmune myocarditis events, and the human subject data was used to extract demographic parameters, co-occurring adverse events (AEs), progression to myocarditis and other variables to characterize the irAE.

### FDA adverse event reporting system (FAERS/AERS) and MedWatch

FAERS/AERS is a database for post marketing safety surveillance reports. It is supported by the United States Food and Drug Administration (FDA). The reporting of AEs and outcomes to FAERS/AERS is done through the MedWatch^44^ platform, predominantly on a voluntary basis. In cases when the reports are submitted to the manufacturer, the manufacturer is mandated to forward the report to FAERS/AERS.

At the time of the study FAERS/AERS contained over fourteen million reports from the first quarter of 2004, which includes reports from prior years, to the second quarter of 2020. The reports were used to run a retrospective analysis of the biologics of interest.

FAERS/AERS data sets are available online at: http://www.fda.gov/Drugs/GuidanceComplianceRegulatoryInformation/Surveillance/AdverseDrugEffects/ucm082193.htm.

### Data preparation

Due to ongoing updates and changes of the format, the quarterly data sets were not all uniform. It was necessary to modify and standardize the sets, creating a consistent uniform table structure with missing columns filled with blanks. Since FAERS/AERS included reports from all over the world many of the variables including drug names were entered according to the country specific generic and brand names and spellings. Online databases were consulted to translate all drug names into single generic terms. Around 0.4% of all the FAERS/AERS reports were duplicates^45–47^. These are either repeated AEs in the same patient or multiple entrances for the same AE occurrence. The duplicates were identified and deleted.

### Study outcomes

The outcome of interest was defined as an adverse event of myocarditis, immune mediated myocarditis, or autoimmune myocarditis. Infection related myocarditis terms such as viral, bacterial, and fungal myocarditis were excluded from the analysis.

### Cohort selection

At the time of the analysis the public database of FAERS/AERS contained 14,202,841 total reports. The following cohorts were compiled for ICI patients, (1) *monotherapy:* ipilimumab (n = 8267), nivolumab (n = 27,149), pembrolizumab (n = 13,476), cemiplimab (n = 161), atezolizumab (n = 2397), avelumab (n = 305), and durvalumab (n = 1710); (2) *anti-PD-1/CTLA-4 combinations:* ipilimumab + nivolumab (n = 7970), ipilimumab + pembrolizumab (n = 225); (3) *ICI* + *axitinib*: pembrolizumab + axitinib (n = 207), avelumab + axitinib (n = 94).

**Figure 1:**
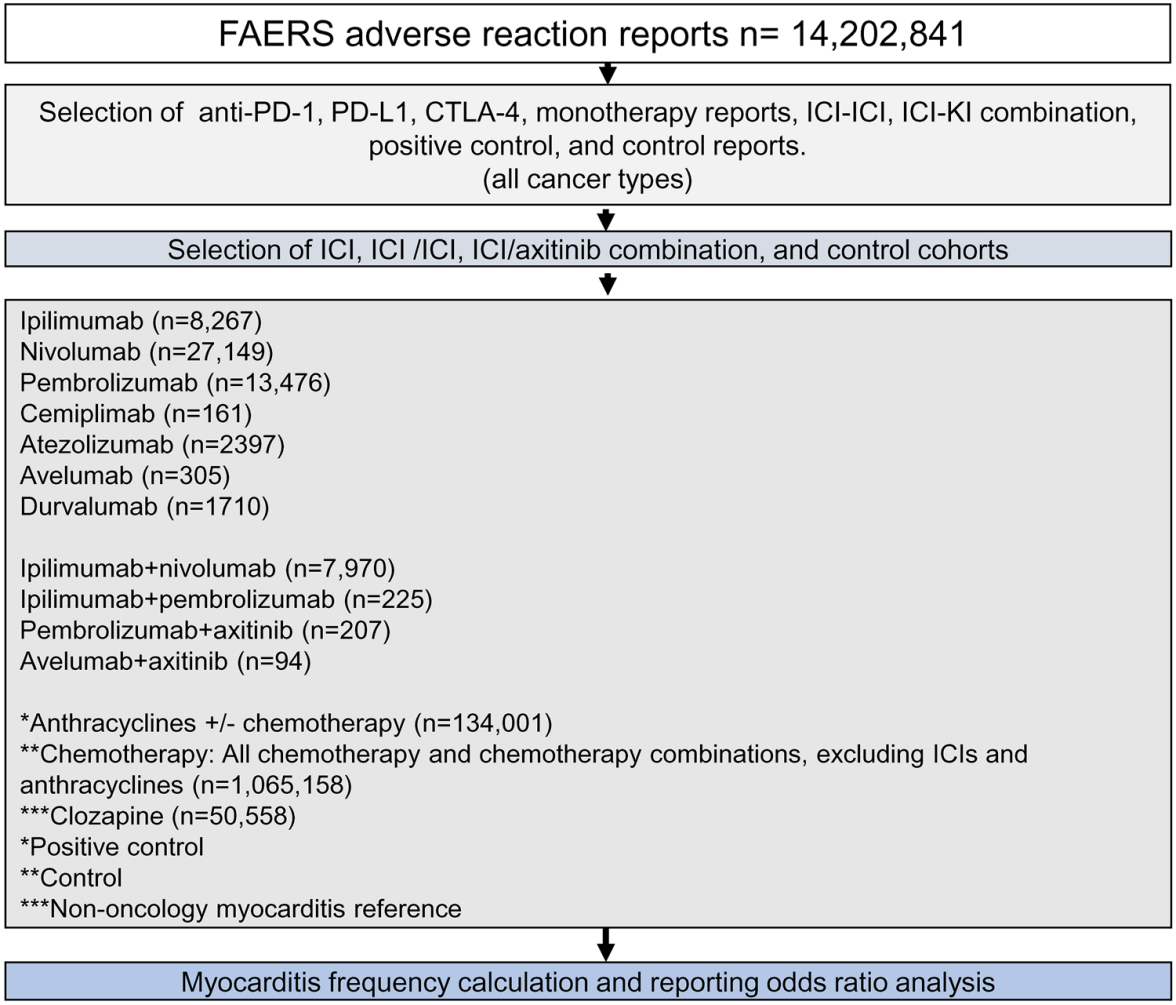
Selection of initial cohorts for ICI monotherapy, anti-PD-1 + CTLA-4, ICI + KI, positive control, and control cohorts.

The following cohorts were compiled as positive controls, and controls used for reporting odds ratio calculation (ROR-controls), (1) *positive control*: anthracyclines with or without chemotherapy (n = 134,001), (2) *ROR-control*: chemotherapy regimens, excluding ICIs and anthracyclines (n = 1,065,158) (Fig. 1 and Supplementary Table S1). Anthracyclines were analyzed separate from chemotherapy as a positive control, since they have been historically associated with myocarditis adverse events^48–50^. Additionally, myocarditis occurrence in FAERS/AERS was calculated for clozapine monotherapy reports as a non-oncology reference point due to its known association with the myocarditis^51–53^. Reported frequencies for the myocarditis reports in the listed cohorts were calculated for an odds ratio analysis to estimate statistical significance of increased reporting. Anthracyclines with or without chemotherapy, rather than clozapine, were chosen as the positive control to preserve oncology indication uniform in the cohorts.

**Figure 2:**
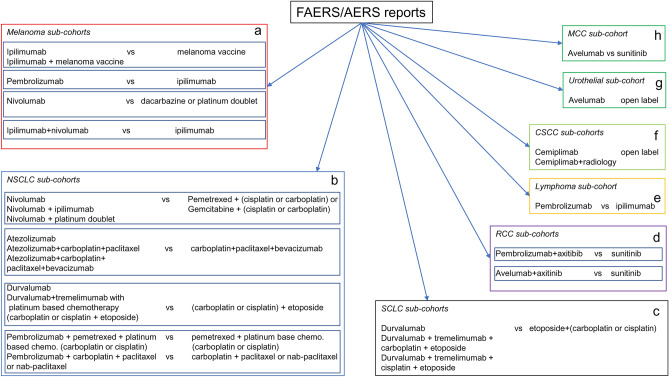
FAERS/AERS cohorts based on indications and treatments in efficacy trials for all ICIs. The two groups on each sub-cohort indicate the treatment *vs* control used in the efficacy trials. *NSCLC* non-small cell lung cancer, *SCLC* small cell lung cancer, *RCC* renal cell carcinoma, *MCC* Merkel cell carcinoma, *CSCC* cutaneous squamous cell carcinoma.

To investigate indication specific myocarditis reports in FAERS/AERS, a separate set of cohorts was created based on indications and treatments in efficacy trials all ICIs (Fig. 2 and Supplementary Table S2): (1) *Melanoma*—ipilimumab (n = 4659), ipilimumab + melanoma vaccine (n = 5), melanoma vaccine (n = 602), pembrolizumab (n = 2686), nivolumab (n = 3239), nivolumab + ipilimumab (n = 3493), dacarbazine (n = 83), carboplatin + paclitaxel (n = 35), (2) *NSCLC*—nivolumab (n = 9432), nivolumab + ipilimumab (n = 511), nivolumab + platinum doublet (n = 155), control-platinum doublet (n = 1564), atezolizumab (n = 1098), atezolizumab + carboplatin + paclitaxel (n = 52), atezolizumab + carboplatin + paclitaxel + bevacizumab (n = 207), carboplatin + paclitaxel + bevacizumab (n = 724), durvalumab (n = 1278), durvalumab + tremelimumab + (carboplatin or cisplatin) + etoposide (n = 17), (carboplatin or cisplatin) + etoposide (n = 344), pembrolizumab + pemetrexed + (carboplatin or cisplatin) (n = 892), pemetrexed + (carboplatin or cisplatin) (n = 1292), pembrolizumab + carboplatin + paclitaxel (n = 616), carboplatin + paclitaxel (n = 1299), (3) *SCLC—*durvalumab (n = 6), durvalumab + tremelimumab + (carboplatin or cisplatin) + etoposide (n = 220), (4) *RCC*—pembrolizumab + axitinib (n = 163), avelumab + axitinib (n = 52), sunitinib (13,624), (5) *Lymphoma*—pembrolizumab (n = 98), (6) *CSCC*—cemiplimab (n = 122), cemiplimab + radiology (n = 1), (7) *Urothelial carcinoma*—avelumab (n = 3), (8) *Merkel call carcinoma*—avelumab (n = 127).

## Results

### Myocarditis event cases in ISS

There were 16 cases of myocarditis out of 20,062 ICI subjects and 1 case out of 4505 in a control/chemotherapy group in ISS. The myocarditis odds ratio (OR) calculation shows an elevation for the ICI group (OR 3.6), though this observation was not statistically significant (95% CI [0.5–27.1]).

**Figure 3:**
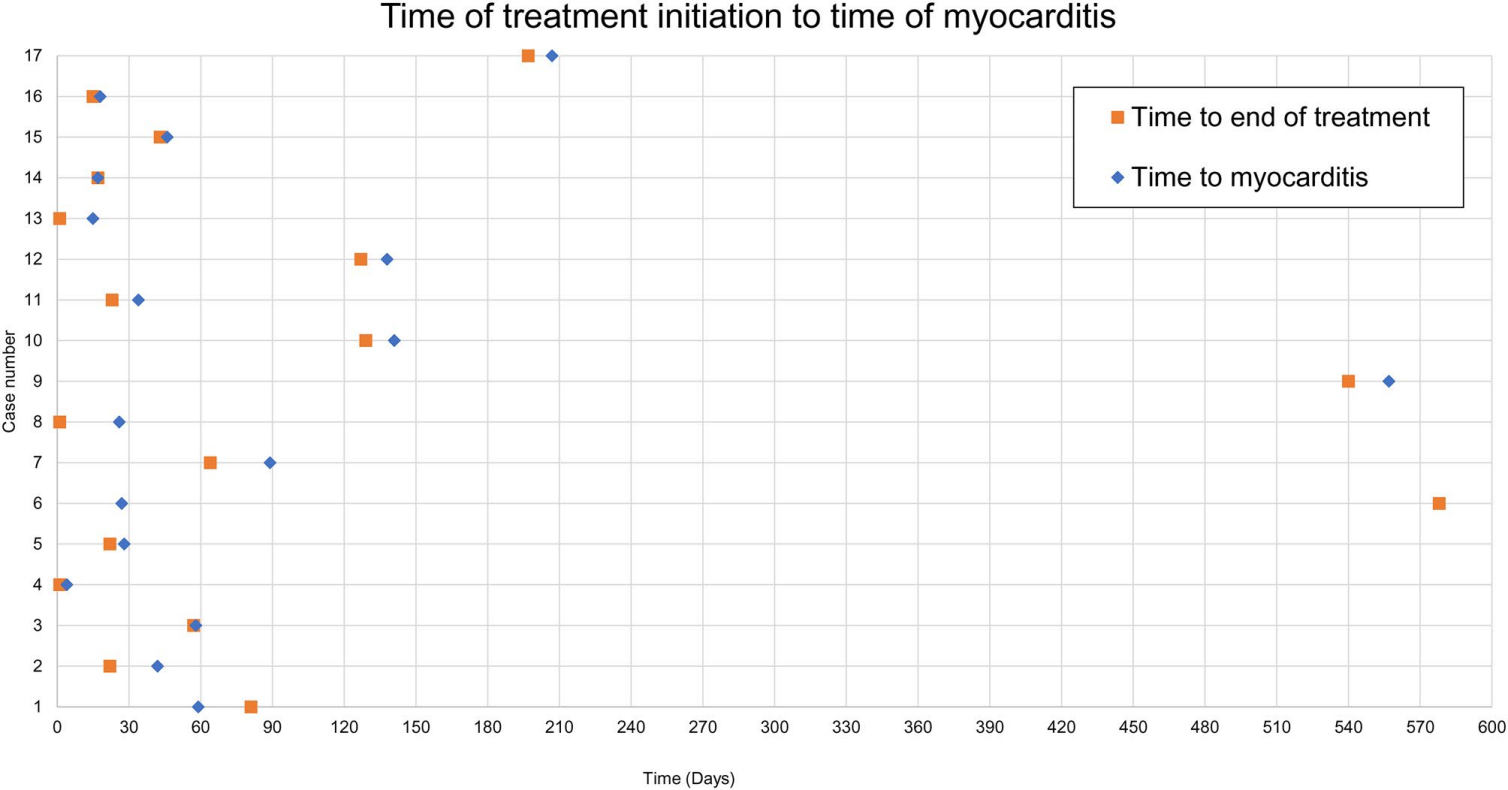
Visualization of the time to end of treatment due to an AE and time to myocarditis X-axis represents the cases numbered in Table 2.

Interestingly, in 14 out of 16 patients who developed myocarditis with ICI, the complication occurred after discontinuation of therapy, independent of overall treatment duration which ranged from 1 to 578 days (Table 2 and Fig. 3).

**Table 2:** Case series.

Subject number	Treatment (dose)^54^	Indication	Treatment duration	Time to myocarditis onset after first drug exposure	Adverse events by grade				
					Grade 1 AE	Grade 2 AE	Grade 3 AE	Grade 4 AE	Grade 5 AE (resulting in death)
1	Carboplatin AUC of 6 mg.mL/min q3w + + pemetrexed 500 mg/m^2^ q3w	*NCSLC*	81 days	59 days	Conjunctivitis Cough Dysgeusia Dysgeusia Dyspnea Herpes simplex Mucosal inflammation Nausea Pyrexia Rash Tachycardia	Dyspepsia Fatigue Lethargy	Myocarditis	None reported	None reported
2	Atezolizumab 1200 mg q3w + cisplatin 75 mg/m^2^ q3w + pemetrexed 500 mg/m^2^ q3w	*NCSLC*	22 days	42 days	C-reactive protein increased Oxygen saturation decreased	None reported	Decreased appetite General physical health deterioration Myocarditis Transient ischemic attack	Lung infection	None reported
3	Cemiplimab (3 mg/kg Q2W)	CSCC	57 days	58 days	Alanine aminotransferase increased Aspartate aminotransferase increased Back pain Blood alkaline phosphatase increased Blood creatine phosphokinase increased Blood creatine phosphokinase MB increased Oral contusion Sensitivity to weather change	Conjunctivitis Eye contusion Eye swelling Visual impairment	Myocarditis	None reported	None reported
4	Durvalumab (10 mg/kg Q2W)	Listed as SCLC and solid tumors	1 day	4 days	Abdominal pain [TR] Ascites [TR] (DW) Back pain Nausea [TR] Oedema peripheral [TR] Troponin increased [TR] Vomiting [TR]	Ascites Dyspnea [TR] Fatigue [TR] Hyperglycemia Myocarditis	Myocarditis Pancreatic carcinoma Troponin increased [TR]	None reported	Pancreatic carcinoma
5	Ipilimumab (10 mg/kg q3w)	Melanoma	22 days	28 days	Diarrhea [+++] Fatigue Musculoskeletal pain Pain in extremity	Cholecystitis Groin pain Hemoglobin decreased Injection-site reaction Periarthritis Pyrexia Allergic rhinitis [+ +]	Hepatitis [+++] (DW) Myocarditis [+++] (DW) Pneumonitis [+++] (DW)	None reported	None reported
6	Ipilimumab (1 mg/kg)	Melanoma	578 days	27 days	Sinus bradycardia [+] (DR) Ventricular extrasystoles [+] (DR)	Blood creatinine increase (DR) Confusional state (DR)	Supraventricular arrhythmia Aspartate aminotransferase increased Blood bilirubin increased Colitis [++] Diarrhea [+] (DW) Hypophosphatemia Hypotension (DR) Leukopenia Lymphopenia Myocarditis	None reported	None reported
7	Ipilimumab (1 mg/kg) + nivolumab (3 mg/kg)	Bladder cancer	64 days	89 days	Acute kidney injury Alanine aminotransferase increased [TR] Anemia [TR] Aspartate aminotransferase increased [TR] Blood albumin decreased Blood alkaline phosphatase increased Blood calcium decreased Blood creatine increased Blood phosphorus decreased Blood urea increased Depression Dry mouth [TR] Dry skin [TR] Hyperthyroidism [TR] Hypomagnesaemia Hypomagnesaemia Edema peripheral Oral candidiasis [TR] Pelvic pain Troponin I increased [TR] Tumor hemorrhage Weight decreased	Angina pectoris [TR] Blood bicarbonate increased [TR] Blood creatine phosphokinase increased [TR] Blood creatine phosphokinase MB increased [TR] Blood gases abnormal [TR] Blood lactic acid decreased Blood lactic acid decreased Blood potassium increased Carbon dioxide increased [TR] Dysgeusia [TR] Escherichia infection Lymphocyte count decreased Malaise [TR] Nausea Neutrophil count increased [TR] Pelvic pain Urinary tract infection Weight decreased	Angina pectoris [TR] Blood creatine phosphokinase increased [TR] Blood creatine phosphokinase MB increased [TR] Constipation [TR] Dry mouth [TR] Dyspnea Myocarditis [TR] Nausea Oral candidiasis [TR] Pelvic pain Stridor [TR] Troponin I increased [TR] Vomiting [TR] (DD)	Myocarditis [TR]	Malignant neoplasm progression
8	Pembrolizumab (2 mg/kg Q3W)	MCC	1 day	26 days	Anemia [TR] Asthenia [TR] Bundle branch block left [TR] Burning sensation [TR] Delirium [TR] Disorientation [TR] Dizziness [TR] Eyelid ptosis [TR] Fall [TR] Leukocytosis [TR] Ophthalmoplegia [TR] Oral candidiasis [TR] Proteinuria [TR]	Acute kidney injury [TR] Atrial fibrillation [TR] Fatigue [TR] Hypertension [TR] Malnutrition [TR]	Acute myocardial infarction [TR] Alanine aminotransferase increased [TR] Aspartate aminotransferase increased [TR] Blood creatine phosphokinase increased [TR] Cardiac failure acute [TR] Encephalopathy [TR] Hyponatremia [TR] Ventricular arrhythmia [TR] Ventricular tachycardia [TR]	Hyperglycemia [TR] Myocarditis [TR] Small intestinal hemorrhage [TR]	None reported
9	Pembrolizumab (200 mg Q3W)	NSCLC	540 days	557 days	Abdominal pain upper[TR] Alanine aminotransferase increased [TR] Aspartate aminotransferase increased [TR] Blood alkaline phosphatase increased [TR] Cardiac failure [TR] Cough Decreased appetite Dyspnea Eczema [TR] Pruritus [TR]	Cough Diarrhea Papule	Acidosis Myocarditis [TR]	None reported	None reported
10	Pembrolizumab (200 mg Q3W)	Bladder cancer	129 days	141 days	Atrioventricular block first degree Blood alkaline phosphatase increased Blood bilirubin increased Bone pain Decreased appetite Fatigue Lymphadenopathy Pleural effusion Pruritus	None reported	Hepatic enzyme increased [TR] Myocarditis [TR] Scrotal oedema	None reported	None reported
11	Pembrolizumab (200 mg Q3W)	Bladder cancer	23 days	34 days	Blood thyroid stimulating hormone increased [TR]	None reported	Back pain [TR] Eyelid ptosis [TR] Fatigue [TR] Hepatitis [TR] Pneumonia [TR] Thyroiditis [TR]	Myocarditis [TR]	Myositis [TR]
12	Pembrolizumab (200 mg Q3W)	Melanoma	127 days	138 days	Weight decreased [TR]	Iodine deficiency	Myocarditis [TR] Myocarditis [TR]	None reported	None reported
13	Pembrolizumab (200 mg Q3W)	HL	1 day	15 days	Diarrhea [TR] Headache [TR]	Tachycardia [TR] Thrombocytopenia Transaminases increased [TR]	Bacteremia Dyspnea [TR] Myositis [TR] Weight decreased [TR]	Myocarditis [TR]	None reported
14	Pembrolizumab (200 mg Q3W) + Axitinib (5 m BID)	RCC	17 days	17 days	Dysphonia [TR]	Chest pain Fatigue [TR] Musculoskeletal chest pain	None reported	None reported	Myocarditis [TR]
15	Pembrolizumab (200 mg Q3W) + Axitinib (5 m BID)	RCC	43 days	46 days	Clostridium difficile colitis Erythema Insomnia Pneumonia	Diarrhea [TR]	Decreased appetite [TR] Electrolyte imbalance [TR]	Hepatic function abnormal Myocarditis [TR]	None reported
16	Avelumab (20 mg/kg Q2W)	Thymoma	15 days	18 days	Dizziness Pyrexia Weight increased	None reported	Autoimmune disorder [TR] (DW) Blood creatine phosphokinase increased [TR] (DW)	None reported	None reported
17	Avelumab (10 mg/kg Q2W)	Head and neck cancer	197 days	207 days	Fatigue Myocarditis Pleural effusion	Hypothyroidism Myocarditis Pleural effusion	None reported	None reported	None reported

[+++] = Certain AERELL; [++] = Probable AERELL; [+] = Possible AERELL; [TR] = Treatment related, plausibility unspecified.

*DR* dose reduced, *DW* drug withdrawn, *DD* dose delayed. Cases are part of the approval packages for the listed ICIs (see PharmaPendium).

**Figure 4:**
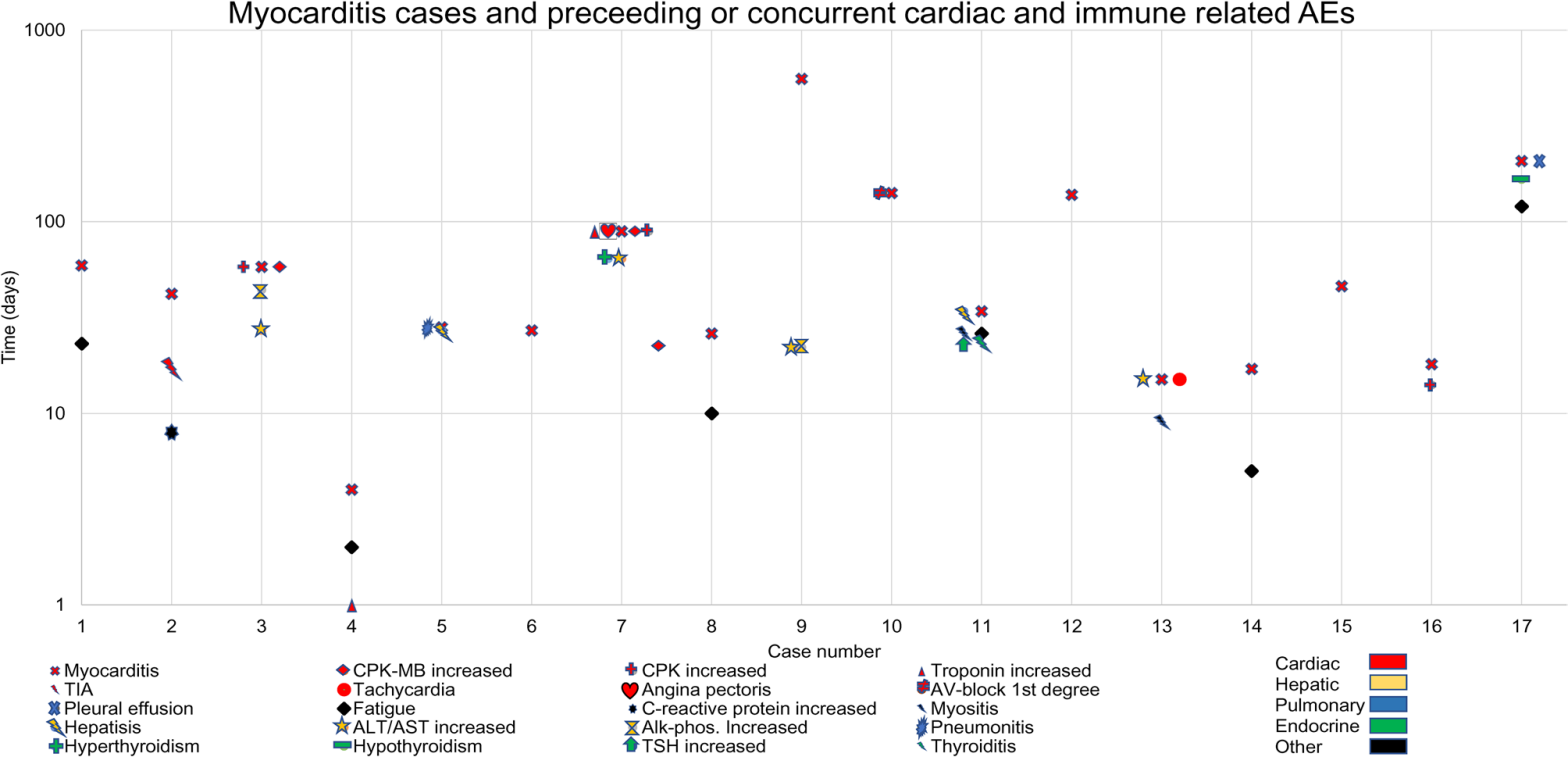
Progression of myocarditis cases with preceding AEs and AEs co-occurring at time of myocarditis.

The most common AEs that occurred prior to and during the onset of myocarditis were cardiac, hepatic, pulmonary, and endocrine irAEs (Table 2, Fig. 4).

### Myocarditis events in FAERS/AERS ICI treatment reports (all cancers)

**Figure 5:**
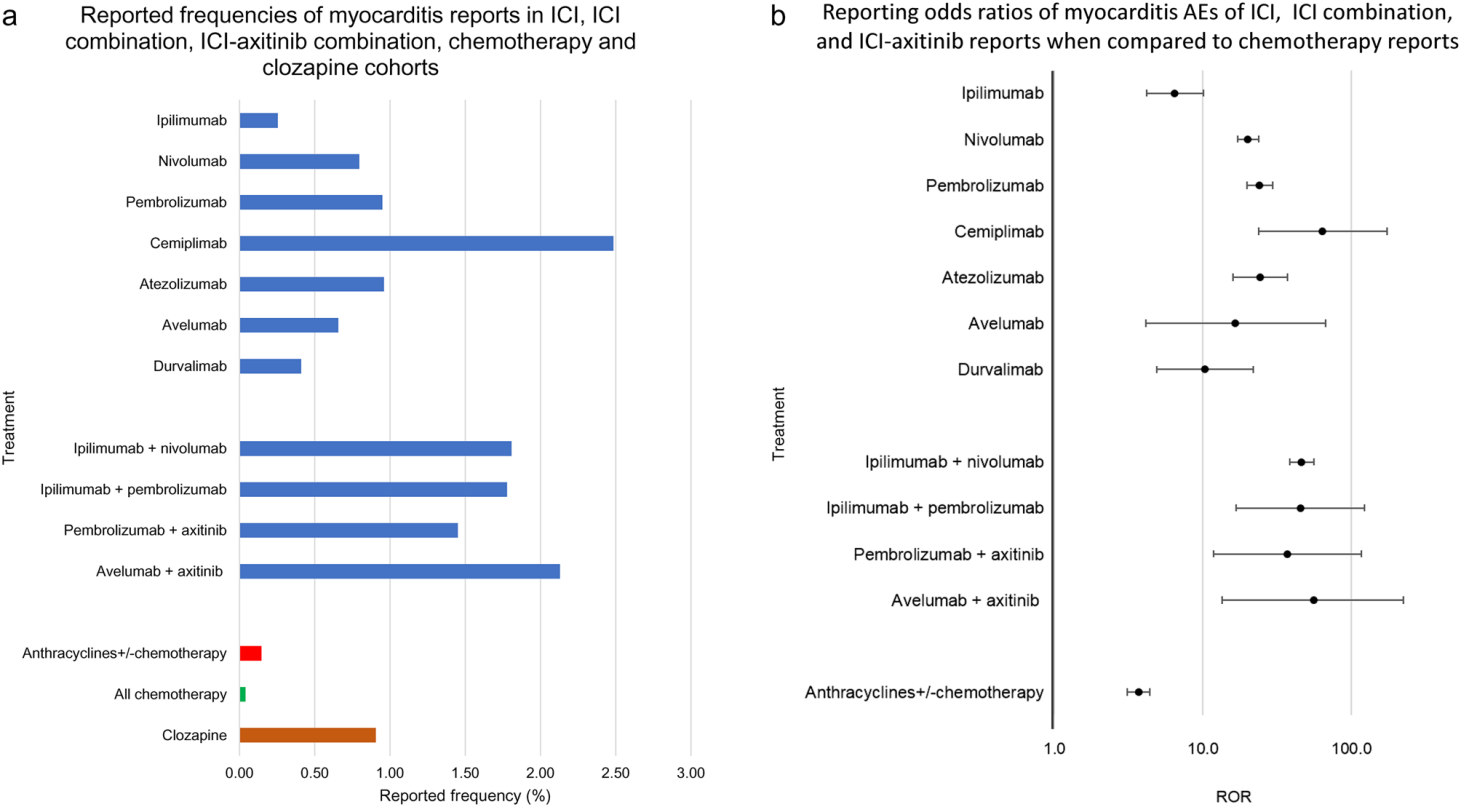
(**a**) Reported frequencies of myocarditis events for patients administered monotherapy: ipilimumab (n = 8267), nivolumab (n = 27,149), pembrolizumab (n = 13,476), cemiplimab (n = 161), atezolizumab (n = 2397), avelumab (n = 305), and durvalumab (n = 1710), ipilimumab + nivolumab (n = 7970), ipilimumab + pembrolizumab (n = 225), pembrolizumab + axitinib (n = 207), avelumab + axitinib (n = 94), anthracyclines with or without chemotherapy (n = 134,001), chemotherapy and chemotherapy combinations, excluding ICIs and anthracyclines (n = 1,065,158), clozapine (n = 50,558. (**b**) Reporting odds ratios were calculated comparing reported frequencies of myocarditis reports in ICI monotherapy, ICI combination and ICI with axitinib cohorts to myocarditis frequencies in chemotherapy cohorts. Anthracyclines ± chemotherapy cohort used as a positive control.

All ICIs were significantly associated with increased reporting myocarditis events: ipilimumab reporting odds ratio (ROR) 6.5, 95% CI [4.2, 10.0], nivolumab (20.1 [17.1, 23.7]), pembrolizumab (24.1 [19.7, 29.4]), cemiplimab (64.0 [23.6, 173.4]), atezolizumab (24.3 [16.0, 37.1]), avelumab (16.6 [4.1, 66.8]), durvalumab (10.3 [4.9, 21.8]). ICI–ICI and ICI–axitinib cohorts had a significant increase in myocarditis reporting compared to ipilimumab, pembrolizumab, nivolumab, or avelumab monotherapy: ipilimumab + nivolumab (46.2 [38.2, 55.9]), ipilimumab + pembrolizumab (45.5 [16.8, 122.7]), pembrolizumab + axitinib (36.9 [11.8, 115.9]), avelumab + axitinib (55.6 [13.4, 222.3]). To compare avelumab and pembrolizumab monotherapy to axitinib monotherapy, the FAERS/AERS database was searched for axitinib monotherapy terms. Interestingly only one report of myocarditis in 5492 axitinib monotherapy reports was found. Anthracyclines ± chemotherapy cohort, selected as a positive control, had a significant association with myocarditis reporting (3.7 [3.1, 4.4]) (Fig. 5). It should be emphasized that the reported frequencies of myocarditis reports (Fig. 5a) and the reporting odds ratios (Fig. 5b) do not represent actual population frequencies, but a statistically significant increased reporting of this irAE to the FAERS/AERS and should be interpreted as such.

### Myocarditis events in FAERS/AERS ICI treatment reports (separated by cancer type)

**Figure 6:**
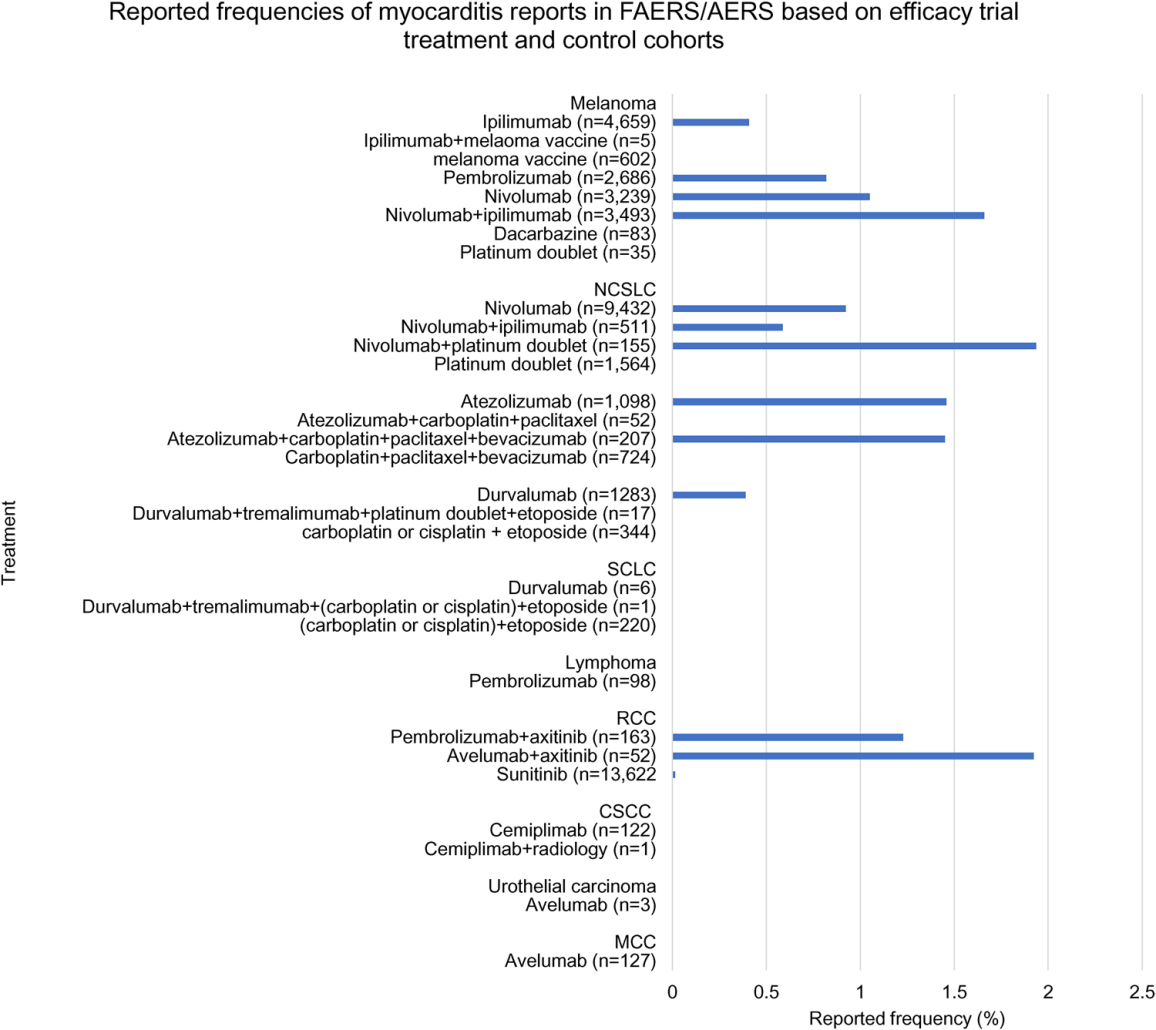
Reported frequencies of myocarditis events in FAERS/AERS database in cohorts based on efficacy trial indications, treatments and control groups. *NSCLC* non-small cell lung cancer, *SCLC* small cell lung cancer, *RCC* renal cell carcinoma, *RCC* renal cell carcinoma, *CSCC* cutaneous squamous cell carcinoma.

When FAERS/AERS reports were organized into cohorts based on the indication, treatment and control groups in the ICI efficacy trials (Table 1 and Fig. 2), myocarditis reports were present in nearly all indication cohorts. There were no reports of myocarditis in any of the control chemotherapy cohorts, thus the statistical difference between cohorts was not evaluated (Fig. 6). The observed trend was similar to the analysis done with all cancer types. ICI–ICI combination, ICI–chemotherapy combination, and ICI–kinase inhibitor combinations had a higher number of myocarditis reporting compared to ICI monotherapy.

The separate dataset with differentiated cohorts by individual ICIs and types of cancer was further analyzed for co-occurring AEs and for death by any cause outcomes. Co-occurring AE analysis was performed for each individual myocarditis case in FAERS/AERS (Supplementary Table S3). 39.9% of all myocarditis cases were associated with death by any cause (Supplementary Table S4). Additionally, there was a significant overlap with the co-occurring AEs observed in the case series (Table 2, Fig. 4, Supplementary S4). Noteworthy was the co-occurrence of myocarditis with myositis (17.6%) and myasthenia gravis (8.2%), in addition to cardiac failure (10.4%), pneumonitis (4, 7), and increased troponin (4.3%) (Supplementary Table S5).

## Discussion

In this study, we evaluated the myocarditis cases in the clinical trials for ICIs, using the ISS^42^ data submitted to the FDA, and the FDA FAERS/AERS database for reports in patients receiving ICIs as monotherapy, in ICI–ICI combinations, ICI in combinations with chemotherapy regimens, and in combinations with axitinib. This is the first comprehensive analysis of the ISS^42^ reports of ICIs. We found that the development of myocarditis occurred earliest on day 4 and the latest on day 557, with a median of 38 days, and this result is consistent with findings in the literature that report that the majority of cases of myocarditis present approximately one to two months after ICI initiation^55^. Most notably, myocarditis occurred 11 days (range 1–25 days) *after* the ICI was discontinued; given that the elimination half-lives of ICIs range from 6.1 days (avelumab) to 27.3 days (pembrolizumab), continued vigilance and monitoring of irAEs is critical for patient safety, well after the ICI is discontinued^56^. The delay in discontinuation of the ICI after myocarditis occurrence may be attributed to the challenges related to diagnosing this rare irAE. Although it takes time to diagnose some of the more complex adverse events, in ISS/ADAE these events are marked/recorded with the date when they were first noticed or suspected, independent of definitive diagnosis date. Since the true therapeutic target of ICI is a T-cell, and irAEs likely represent endogenous immunologic phenomena, it is possible that the administration of ICI leading to immune myocarditis may not have been the most proximal administration to the time of symptomatic deterioration.

Of note, the myocarditis incidence of 0.08% seen in the ISS data, is consistent with prior studies among patients receiving ICIs which have demonstrated an incidence of myocarditis ranging from 0.04 to 1.14%^31,36,39,55,57,58^. Deducing a statistically significant conclusion regarding associations between disease histology or various therapies and the development of myocarditis is limited by the small number of the ISS myocarditis cases.

By analyzing the FDA FAERS/AERS database, we confirmed the association between myocarditis and ICIs with the frequency of myocarditis ranging from 0.25 to 2.48% in patients receiving ICIs as monotherapy, in combination with ICI, in combination with axitinib, and in combination with chemotherapy regimens. We demonstrated the association of myocarditis was stronger with combined ICIs as compared to ICI monotherapy which in agreement with prior studies^35,59^; patients receiving ipilimumab with nivolumab demonstrated the highest reported risk of myocarditis. The all-cause reported mortality rate was 39.9% in all the ICI patients who experienced myocarditis. Melanoma patients with myocarditis due to ipilimumab with nivolumab use had a higher rate of all-cause mortality (53.4%) compared to ICI monotherapy (Supplementary Table S4)^35,59^.

In contrast to the disproportionality and Bayesian analyses completed by Fan et al.^35^, we determined that avelumab monotherapy did not have as strong of an association with myocarditis, while avelumab combined with axitinib did demonstrate a stronger association. The disparity in the results may be due to the fact that our analysis included ICI monotherapy reports, while Fan and colleagues used ICI reports where concomitant drugs were used and ICI was the primary suspect which allows for significant bias and error, especially when searching for a rare or unexpected event. Furthermore, we organized the reports into cohorts by specific indication, and quantified the myocarditis association by using positive and negative controls with the same type of cancer, which is rarely done in disproportionality analysis studies. Additionally, we analyzed the co-occurring adverse events and observed myositis to be the most common AE reported with myocarditis, suggesting a stronger etiological connection between the two irAEs that may not be explained by general immune activation alone. The association between myocarditis and myositis has been previously noted in previous immunotherapy^60,61^, autoimmune disease^62,63^, and infection case studies^64,65^.

Taken together, the results from the ISS and the FDA FAERS/AERS databases highlight the need for prompt recognition, diagnosis, and management of myocarditis in patients receiving ICIs, with additional vigilance with ICIs combination therapies, from treatment initiation through several weeks *after* ICI discontinuation.

The monitoring and management of cardiac irAEs has been well described by Palaskas and Spallarosa; a diagnostic workup including the use of laboratory values (Troponin I, N-terminal pro B-type natriuretic (BNP) peptide, BNP), imaging (12-lead electrocardiogram, echocardiogram, cardiac magnetic resonance, telemetry monitoring), and procedures (endomyocardial biopsy and coronary angiography) is recommended in patients with suspected myocarditis^55,66^. While the diagnostic workup is performed, ICI therapy should be discontinued, and prompt initiation of corticosteroids (1000 mg intravenous (IV) methylprednisolone for three days followed by 1 mg/kg IV/oral prednisone) is recommended. If the diagnostic workup demonstrates definite, probable, or possible myocarditis, corticosteroids should be continued and tapered off over four to six weeks. Of particular importance is attention to the electrocardiographic changes that occur in myocarditis, such as arrythmias^67–69^ is the predominant mechanism of morbidity and mortality and close consultation with cardiology colleagues and in particular electrophysiology subspecialists is key in the multidisciplinary care of these patients.

In summary, we confirmed statistically significant association of ICI use with myocarditis using FARS/AERS data and stratified this association by specific cancer types and by ICI combination therapies. We found and an increased reporting of myocarditis cases for patients treated with ICI–ICI, ICI–axitinib, and ICI–chemotherapy combinations.

### Study limitations

Adverse event reporting to FAERS/AERS is voluntary and reports are not always clinically adjudicated for causality. The calculated frequencies do not represent the actual population but rather the *reported frequency* of myocarditis AEs out of all reported ICI AEs in the FAERS/AERS database. This *reported frequency* definition needs to be kept in mind while evaluating those frequencies, as the numbers may be exaggerated and do not represent the actual number of cases in the total ICI-administered population. Studies have shown that there may be significant underreporting and overreporting of adverse events^70,71^. Absence of lab values and complete medical records, including comprehensive information, concurrent medications, presence of a pacemaker, and comorbidities may introduce uncertainties to our analysis. However, using postmarketing surveillance data remains an important tool in identifying a statistically significant signal, especially for very rare adverse events such as myocarditis which was virtually non-existent in the clinical trial data. Additionally, both in ISS and FAERS/AERS data, a noteworthy limitation is the variability in the way the adverse events were coded. There is a need for harmonization of nomenclature, a consensus, and having the ability for algorithms to cluster terms to better understanding temporal kinetics.

## Supplementary Information

Supplementary Tables.

## Data Availability

There was no direct human participation in this study. The data sets utilized were de-identified. Institutional Review Board requirements do not apply under 45 CFR 46.102. Cases used in the case-series section were included in the approval package: https://www.pharmapendium.com. FAERS/AERS datasets are available to the public online: https://www.fda.gov/drugs/questions-and-answers-fdas-adverse-event-reporting-system-faers/fda-adverse-event-reporting-system-faers-latest-quarterly-data-files.

## References

[1] Assal A, Kaner J, Pendurti G, Zang X (2015). Emerging targets in cancer immunotherapy: Beyond CTLA-4 and PD-1. Immunotherapy.

[2] Alexander W (2016). The checkpoint immunotherapy revolution: What started as a trickle has become a flood, despite some daunting adverse effects; new drugs, indications, and combinations continue to emerge. P T..

[3] Spiers L, Coupe N, Payne M (2019). Toxicities associated with checkpoint inhibitors-an overview. Rheumatology (Oxford).

[4] Bajwa R, Cheema A, Khan T (2019). Adverse effects of immune checkpoint inhibitors (programmed death-1 inhibitors and cytotoxic T-lymphocyte-associated protein-4 inhibitors): Results of a retrospective study. J. Clin. Med. Res..

[5] Yervoy Highlights of Prescribing Information, https://www.accessdata.fda.gov/drugsatfda_docs/label/2020/125377s110lbl.pdf (2020).

[6] Keytruda Highlights of Prescribing Information, https://www.accessdata.fda.gov/drugsatfda_docs/label/2021/125514s096lbl.pdf (2021).

[7] Libtayo Highlights of Prescribing Information, https://www.accessdata.fda.gov/drugsatfda_docs/label/2021/761097s008lbl.pdf (2021).

[8] Tecentriq Highlights of Prescribing Information, https://www.accessdata.fda.gov/drugsatfda_docs/label/2020/761034s028lbl.pdf (2020).

[9] Imfinzi Highlights of Prescribing Information, https://www.accessdata.fda.gov/drugsatfda_docs/label/2018/761069s002lbl.pdf (2018).

[10] Bavencio Highlights of Prescribing Information. EMD Serono, Inc.2020.

[11] Motzer RJ, Penkov K, Haanen J (2019). Avelumab plus axitinib versus sunitinib for advanced renal-cell carcinoma. N. Engl. J. Med..

[12] MDX-010 Antibody, MDX-1379 Melanoma Vaccine, or MDX-010/MDX-1379 combination treatment for patients with unresectable or metastatic melanoma, https://www.clinicaltrials.gov/ct2/show/NCT00094653 (2011).

[13] Hellmann MD, Paz-Ares L, Bernabe Caro R (2019). Nivolumab plus ipilimumab in advanced non-small-cell lung cancer. N. Engl. J. Med..

[14] Robert C, Schachter J, Long GV (2015). Pembrolizumab versus ipilimumab in advanced melanoma. N. Engl. J. Med..

[15] Chen R, Zinzani PL, Fanale MA (2017). Phase II study of the efficacy and safety of pembrolizumab for relapsed/refractory classic hodgkin lymphoma. J. Clin. Oncol..

[16] Rini BI, Plimack ER, Stus V (2019). Pembrolizumab plus axitinib versus sunitinib for advanced renal-cell carcinoma. N. Engl. J. Med..

[17] Gandhi L, Rodríguez-Abreu D, Gadgeel S (2018). Pembrolizumab plus chemotherapy in metastatic non-small-cell lung cancer. N. Engl. J. Med..

[18] Paz-Ares L, Luft A, Vicente D (2018). Pembrolizumab plus chemotherapy for squamous non-small-cell lung cancer. N. Engl. J. Med..

[19] Opdivo Highlights of Prescribing Information, https://www.accessdata.fda.gov/drugsatfda_docs/label/2021/125554s090lbl.pdf (2021).

[20] Weber JS, D'Angelo SP, Minor D (2015). Nivolumab versus chemotherapy in patients with advanced melanoma who progressed after anti-CTLA-4 treatment (CheckMate 037): A randomised, controlled, open-label, phase 3 trial. Lancet Oncol..

[21] Migden MR, Rischin D, Schmults CD (2018). PD-1 blockade with cemiplimab in advanced cutaneous squamous-cell carcinoma. N. Engl. J. Med..

[22] Balar AV, Galsky MD, Rosenberg JE (2017). Atezolizumab as first-line treatment in cisplatin-ineligible patients with locally advanced and metastatic urothelial carcinoma: A single-arm, multicentre, phase 2 trial. Lancet.

[23] Socinski MA, Jotte RM, Cappuzzo F (2018). Atezolizumab for first-line treatment of metastatic nonsquamous NSCLC. N. Engl. J. Med..

[24] A Phase 1/2 Study to Evaluate MEDI4736, https://clinicaltrials.gov/ct2/show/NCT01693562 (2011).

[25] Antonia SJ, Villegas A, Daniel D (2017). Durvalumab after chemoradiotherapy in stage III non-small-cell lung cancer. N. Engl. J. Med..

[26] Paz-Ares L, Dvorkin M, Chen Y (2019). Durvalumab plus platinum-etoposide versus platinum-etoposide in first-line treatment of extensive-stage small-cell lung cancer (CASPIAN): A randomised, controlled, open-label, phase 3 trial. Lancet.

[27] D'Angelo SP, Russell J, Lebbé C (2018). Efficacy and safety of first-line avelumab treatment in patients with stage IV metastatic merkel cell carcinoma: A preplanned interim analysis of a clinical trial. JAMA Oncol..

[28] Hassan R, Thomas A, Nemunaitis JJ (2019). Efficacy and safety of avelumab treatment in patients with advanced unresectable mesothelioma: Phase 1b results from the JAVELIN solid tumor trial. JAMA Oncol..

[29] Leone O, Pieroni M, Rapezzi C, Olivotto I (2019). The spectrum of myocarditis: From pathology to the clinics. Virchows Arch..

[30] Sagar S, Liu PP, Cooper LT (2012). Myocarditis. Lancet.

[31] Moslehi JJ, Salem JE, Sosman JA, Lebrun-Vignes B, Johnson DB (2018). Increased reporting of fatal immune checkpoint inhibitor-associated myocarditis. Lancet.

[32] Wang DY, Salem JE, Cohen JV (2018). Fatal toxic effects associated with immune checkpoint inhibitors: A systematic review and meta-analysis. JAMA Oncol..

[33] Master SR, Robinson A, Mills GM, Mansour RP (2019). Cardiovascular complications of immune checkpoint inhibitor therapy. J. Clin. Oncol..

[34] Wang F, Sun X, Qin S (2020). A retrospective study of immune checkpoint inhibitor-associated myocarditis in a single center in China. Chin. Clin. Oncol..

[35] Fan Q, Hu Y, Yang C, Zhao B (2019). Myocarditis following the use of different immune checkpoint inhibitor regimens: A real-world analysis of post-marketing surveillance data. Int. Immunopharmacol..

[36] Johnson DB, Balko JM, Compton ML (2016). Fulminant myocarditis with combination immune checkpoint blockade. N. Engl. J. Med..

[37] Saibil SD, Bonilla L, Majeed H (2019). Fatal myocarditis and rhabdomyositis in a patient with stage IV melanoma treated with combined ipilimumab and nivolumab. Curr. Oncol..

[38] Zamami Y, Niimura T, Okada N (2019). Factors associated with immune checkpoint inhibitor-related myocarditis. JAMA Oncol..

[39] Mahmood SS, Fradley MG, Cohen JV (2018). Myocarditis in patients treated with immune checkpoint inhibitors. J. Am. Coll. Cardiol..

[40] Heinzerling L, Ott PA, Hodi FS (2016). Cardiotoxicity associated with CTLA4 and PD1 blocking immunotherapy. J. Immunother. Cancer..

[41] Trotti A, Colevas AD, Setser A (2003). CTCAE v3.0: Development of a comprehensive grading system for the adverse effects of cancer treatment. Semin. Radiat. Oncol..

[42] United States Food and Drug Administration. Placement of Integrated Summaries of Safety and Effectiveness (ISS/ISE) in Applications Submitted in the eCTD Format, https://www.fda.gov/drugs/electronic-regulatory-submission-and-review/placementintegrated-summaries-safety-and-effectiveness-issise-applications-submitted-ectd-format (Accessed Jan. 2020).

[43] Center for Drug Evaluation and Research. CDER System of Record. Unites States Food and Drug Administration, https://www.fda.gov/media/89742/download (Accessed Dec. 2019)

[44] Craigle V (2007). MedWatch: The FDA safety information and adverse event reporting program. J. Med. Libr. Assoc..

[45] Makunts T, Atayee RS, Abagyan R (2019). Retrospective analysis reveals significant association of hypoglycemia with tramadol and methadone in contrast to other opioids. Sci. Rep..

[46] Makunts T, Alpatty S, Lee KC, Atayee RS, Abagyan R (2019). Proton-pump inhibitor use is associated with a broad spectrum of neurological adverse events including impaired hearing, vision, and memory. Sci. Rep..

[47] Cohen IV, Makunts T, Moumedjian T, Issa MA, Abagyan R (2020). Cardiac adverse events associated with chloroquine and hydroxychloroquine exposure in 20 years of drug safety surveillance reports. Sci. Rep..

[48] Gaudin PB, Hruban RH, Beschorner WE (1993). Myocarditis associated with doxorubicin cardiotoxicity. Am. J. Clin. Pathol..

[49] Jones RL, Swanton C, Ewer MS (2006). Anthracycline cardiotoxicity. Expert. Opin. Drug Saf..

[50] Cai F, Luis MAF, Lin X (2019). Anthracycline-induced cardiotoxicity in the chemotherapy treatment of breast cancer: Preventive strategies and treatment. Mol. Clin. Oncol..

[51] Malik AH, Shetty S, Aronow WS (2019). Clozapine-associated myocarditis: Is it time to start monitoring?. Am. J. Ther..

[52] Siskind D, Sidhu A, Cross J (2020). Systematic review and meta-analysis of rates of clozapine-associated myocarditis and cardiomyopathy. Aust. N. Z. J. Psychiatry..

[53] Haas SJ, Hill R, Krum H (2007). Clozapine-associated myocarditis: A review of 116 cases of suspected myocarditis associated with the use of clozapine in Australia during 1993–2003. Drug Saf..

[54] PharmaPendium. The Essential Drug Safety Resource https://www.pharmapendium.com (accessed 2021).

[55] Palaskas N, Lopez-Mattei J, Durand JB, Iliescu C, Deswal A (2020). Immune checkpoint inhibitor myocarditis: Pathophysiological characteristics, diagnosis, and treatment. J. Am. Heart Assoc..

[56] Centanni M, Moes DJAR, Trocóniz IF, Ciccolini J, van Hasselt JGC (2019). Clinical pharmacokinetics and pharmacodynamics of immune checkpoint inhibitors. Clin. Pharmacokinet..

[57] Salem JE, Manouchehri A, Moey M (2018). Cardiovascular toxicities associated with immune checkpoint inhibitors: An observational, retrospective, pharmacovigilance study. Lancet Oncol..

[58] Al-Kindi SG, Oliveira GH (2018). Reporting of immune checkpoint inhibitor-associated myocarditis. Lancet.

[59] Raschi E, Mazzarella A, Antonazzo IC (2019). Toxicities with immune checkpoint inhibitors: Emerging priorities from disproportionality analysis of the FDA adverse event reporting system. Target Oncol..

[60] Touat M, Maisonobe T, Knauss S (2018). Immune checkpoint inhibitor-related myositis and myocarditis in patients with cancer. Neurology.

[61] Suzuki S, Ishikawa N, Konoeda F (2017). Nivolumab-related myasthenia gravis with myositis and myocarditis in Japan. Neurology.

[62] West SG, Killian PJ, Lawless OJ (1981). Association of myositis and myocarditis in progressive systemic sclerosis. Arthritis Rheum..

[63] Borenstein DG, Fye WB, Arnett FC, Stevens MB (1978). The myocarditis of systemic lupus erythematosus: Association with myositis. Ann. Intern. Med..

[64] Kumar K, Guirgis M, Zieroth S (2011). Influenza myocarditis and myositis: Case presentation and review of the literature. Can. J. Cardiol..

[65] Sangle SA, Dasgupta A, Ratnalikar SD, Kulkarni RV (2010). Dengue myositis and myocarditis. Neurol. India..

[66] Spallarossa P, Sarocchi M, Tini G (2020). How to monitor cardiac complications of immune checkpoint inhibitor therapy. Front. Pharmacol..

[67] Bonaca MP, Olenchock BA, Salem JE (2019). Myocarditis in the setting of cancer therapeutics: Proposed case definitions for emerging clinical syndromes in cardio-oncology. Circulation.

[68] Moslehi JJ (2016). Cardiovascular toxic effects of targeted cancer therapies. N. Engl. J. Med..

[69] Thompson JA, Schneider BJ, Brahmer J (2020). NCCN guidelines insights: Management of immunotherapy-related toxicities, Version 1.2020. J. Natl. Compr. Cancer Netw..

[70] Alatawi YM, Hansen RA (2017). Empirical estimation of under-reporting in the U.S. Food and Drug Administration Adverse Event Reporting System (FAERS). Expert. Opin. Drug Saf..

[71] Maciejewski M, Lounkine E, Whitebread S (2017). Reverse translation of adverse event reports paves the way for de-risking preclinical off-targets. Elife.

