# Supplementary material for: Myocarditis occurrence with cancer immunotherapy across indications in clinical trial and post-marketing data
Source: Sci Rep. 2021 Aug 30;11:17324. doi: 10.1038/s41598-021-96467-5 (PMC8405813; doi:10.1038/s41598-021-96467-5)
Supplement: Supplementary file 1 — Supplementary Tables. [file 41598_2021_96467_MOESM1_ESM.docx]

Myocarditis occurrence with cancer immunotherapy across indications in clinical trial and post-marketing data

Tigran Makunts^1,2^, Ila M. Saunders^2^, Isaac V. Cohen^3^, Mengxing Li^1^, Talar Moumedjian^2^, Masara A. Issa^2^, Keith Burkhart^4^, Peter Lee^4^, Sandip Pravin Patel^5^, Ruben Abagyan^2*^

^1^Oak Ridge Institute of Science and Education fellowship at Office of Clinical Pharmacology, United States Food and Drug Administration

^2^ Skaggs School of Pharmacy and Pharmaceutical Sciences, University of California San Diego, La Jolla, California

^3^Clinical Pharmacology and Therapeutics (CPT) Postdoctoral Training Program, University of California San Francisco, San Francisco, California

^4^Center for Drug Evaluation and Research, US Food and Drug Administration, Silver Spring, Maryland

^5^Division of Hematology and Medical Oncology, UCSD Moores Cancer Center, La Jolla, California

Correspondence to: RA [rabagyan@health.ucsd.edu](mailto:rabagyan@health.ucsd.edu)

**Supplementary information**

**Table S1**

| **Category** | **Drug/Biologic** |
| --- | --- |
| Anti-CTLA4 | Ipilimumab |
| Anti-PD-1 | Pembrolizumab, nivolumab, cemiplimab |
| Anti-PD-L1 | Atezolizumab, avelumab, durvalumab |
| Anthracyclines | Daunorubicin, doxorubicin, epirubicin, idarubicin, mitoxantrone, valrubicin |
| Combinations | Ipilimumab+nivolumab, ipilimumab+pembrolizumab,  pembrolizumab+axitinib, avelumab+axitinib |
| Other chemotherapy | Paclitaxel, docetaxel, vinorbeline, vincristine, vinblastine, pemetrexed, gemcitabine, decitabine, cisplatin, carboplatin, oxaliplatin, nedaplatin, paraplatin, satraplatin, etoposide, teniposide, cyclophosphamide, methotrexate, mercaptopurine, pralatrexate, 5-fluorouracil, eribulin, capecitabine, dacarbazine, procarbazine, temozolamide, dactinomycin, actinomycin, mitomycin, lomustine, fotemustine, estramustine, carmustine, irinotecan, topotecan, cytarabine, |

Table S1. Drug and biologic names used in study cohort selections.

**Table S2**

| **Type of cancer** | **FAERS/AERS terms** |
| --- | --- |
| *Melanoma* | malignant_melanoma  metastatic_malignant_melanoma  malignant_melanoma_stage_iv  malignant_melanoma_stage_iii  intraocular_melanoma  melanoma_recurrent  metastatic_ocular_melanoma  nodular_melanoma  uveal_melanoma  choroid_melanoma  melanoma  desmoplastic_melanoma  acral_lentiginous_melanoma  gastrointestinal_melanoma  malignant_melanoma_stage_ii  malignant_melanoma_of_eyelid  genitourinary_melanoma  conjunctival_melanoma  acral_lentiginous_melanoma_stage_iv  superficial_spreading_melanoma_stage_unspecified  superficial_spreading_melanoma_stage_iv  superficial_spreading_melanoma_stage_iii  metastatic_melanoma  malignant_melanoma_stage_i  malignant_melanoma_in_situ  iris_melanoma  central_nervous_system_melanoma  acral_lentiginous_melanoma_stage_ii |
| *NSCLC* | non_small_cell_lung_cancer  lung_neoplasm_malignant  lung_adenocarcinoma  non_small_cell_lung_cancer_recurrent  squamous_cell_carcinoma_of_lung  lung_adenocarcinoma_stage_iv  lung_cancer_metastatic  non_small_cell_lung_cancer_metastatic  non_small_cell_lung_cancer_stage_iv  lung_carcinoma_cell_type_unspecified_stage_iv  metastases_to_lung  lung_squamous_cell_carcinoma_stage_iv  lung_adenocarcinoma_stage_iii  lung_neoplasm  lung_squamous_cell_carcinoma_stage_iii  lung_adenocarcinoma_metastatic  lung_squamous_cell_carcinoma_metastatic  lung_carcinoma_cell_type_unspecified_stage_0  large_cell_lung_cancer  lung_adenocarcinoma_recurrent  lung_squamous_cell_carcinoma_recurrent  adenosquamous_cell_lung_cancer  non_small_cell_lung_cancer_stage_iiib  non_small_cell_lung_cancer_stage_iiia  lung_carcinoma_cell_type_unspecified_stage_iii  adenosquamous_cell_lung_cancer_stage_iv  lung_carcinoma_cell_type_unspecified_recurrent  lung_adenocarcinoma_stage_ii  non_small_cell_lung_cancer_stage_iii  lung_adenocarcinoma_stage_i  non_small_cell_lung_cancer_stage_ii  lung_squamous_cell_carcinoma_stage_ii  lung_squamous_cell_carcinoma_stage_i  lung_carcinoma_cell_type_unspecified_stage_i  large_cell_lung_cancer_stage_iv  large_cell_lung_cancer_stage_iii  neuroendocrine_tumour_of_the_lung  sarcomatoid_carcinoma_of_the_lung- keep  non_small_cell_lung_cancer_stage_0  neuroendocrine_tumour_of_the_lung_metastatic  non_small_cell_lung_cancer_stage_i  lung_carcinoma_cell_type_unspecified_stage_ii  large_cell_lung_cancer_metastatic  adenosquamous_cell_lung_cancer_stage_iii |
| *SCLC* | small_cell_lung_cancer  small_cell_lung_cancer_recurrent  small_cell_lung_cancer_limited_stage  small_cell_lung_cancer_metastatic  small_cell_lung_cancer_extensive_stage |
| *RCC* | renal_cell_carcinoma  metastatic_renal_cell_carcinoma  renal_cancer  renal_cancer_metastatic  clear_cell_renal_cell_carcinoma  transitional_cell_cancer_of_the_renal_pelvis_and_ureter  malignant_neoplasm_of_renal_pelvis  transitional_cell_cancer_of_renal_pelvis_and_ureter_metastatic  renal_neoplasm  papillary_renal_cell_carcinoma  renal_cell_carcinoma_stage_iv  renal_cancer_stage_iv  non_renal_cell_carcinoma_of_kidney  renal_cell_carcinoma_recurrent  hereditary_leiomyomatosis_renal_cell_carcinoma  renal_cancer_stage_iii  renal_cell_carcinoma_stage_ii |
| *Lymphoma* | diffuse_large_b_cell_lymphoma  lymphoma  non_hodgkin_s_lymphoma  central_nervous_system_lymphoma  b_cell_lymphoma  t_cell_lymphoma  natural_killer_cell_lymphoblastic_lymphoma  peripheral_t_cell_lymphoma_unspecified  primary_mediastinal_large_b_cell_lymphoma  angiocentric_lymphoma  mantle_cell_lymphoma  non_hodgkin_s_lymphoma_refractory  metastatic_lymphoma  marginal_zone_lymphoma  peripheral_t_cell_lymphoma_unspecified_stage_iv  b_cell_lymphoma_stage_iv  b_cell_lymphoma_recurrent  anaplastic_large_cell_lymphoma  b_cell_lymphoma_stage_ii  b_cell_lymphoma_refractory  angioimmunoblastic_t_cell_lymphoma |
| *CSCC* | squamous_cell_carcinoma_of_skin  squamous_cell_carcinoma_of_skin_in_situ  skin_squamous_cell_carcinoma_metastatic  skin_squamous_cell_carcinoma_recurrent |
| *Merkel Cell* | merkel_cell_carcinoma  neuroendocrine_carcinoma_of_the_skin |
| *Urothelial* | urothelial_carcinoma_bladder  urothelial_carcinoma  bladder_cancer_recurrent  bladder_cancer  carcinoma_in_situ_of_bladder  metastatic_carcinoma_of_the_bladder  bladder_transitional_cell_carcinoma_metastatic  bladder_cancer_stage_iv  bladder_cancer_stage_0_with_cancer_in_situ  bladder_transitional_cell_carcinoma_stage_i  bladder_cancer_stage_iii  bladder_transitional_cell_carcinoma_recurrent  bladder_transitional_cell_carcinoma_stage_iv  metastases_to_bladder  bladder_cancer_stage_i_with_cancer_in_situ  bladder_transitional_cell_carcinoma_stage_iii  bladder_transitional_cell_carcinoma_stage_ii  bladder_transitional_cell_carcinoma_stage_0  invasive_bladder_cancer  bladder_carcinoma  urothelial_carcinoma_bladder  bladder_cancer_stage_i_without_cancer_in_situ |

Table S2. FAERS/AERS cancer immunotherapy Indication list used in the analysis.

**Table S3**

| **Indication** | **Treatment** | **Myocarditis case number/total** | **Cooccurring AEs in each individual Myocarditis case** | **Top 10 most common unique cooccurring AEs** | **Death by any cause in myocarditis patients** |
| --- | --- | --- | --- | --- | --- |
| Melanoma | ipilimumab | 19/4659 | acute_kidney_injury;autoimmune_hepatitis;dermatitis;myocarditis;renal_impairment  atrial_fibrillation;colitis;intentional_product_use_issue;myocarditis;off_label_use;troponin_increased  atrial_fibrillation;colitis;myocarditis;off_label_use;product_use_in_unapproved_indication;troponin_increased  autoimmune_hepatitis;autoimmune_myocarditis;death  autoimmune_hepatitis;cardiac_failure_congestive;myocarditis;therapeutic_response_decreased;death  autoimmune_myocarditis  autoimmune_myocarditis;cardiac_failure_congestive  cardiac_failure_congestive;myocarditis;pneumonitis  cardiopulmonary_failure;myocarditis;death  coronary_artery_disease;myocarditis;troponin_increased  diarrhoea;dizziness;myocarditis;death  dyspnoea;myocarditis  hepatitis;myasthenic_syndrome;myocarditis;myositis  myocarditis;death  myocarditis;off_label_use;death  myocarditis;ventricular_tachycardia  atrial_fibrillation;cardiomyopathy;embolism;hypoxia;left_ventricular_dysfunction;myocarditis;pleural_effusion  atrial_fibrillation;cardiomyopathy;diarrhoea;embolism;left_ventricular_dysfunction;myocarditis;pleural_effusion  atrial_fibrillation;cardiomyopathy;diarrhoea;embolism;hypoxia;left_ventricular_dysfunction;myocarditis;pleural_effusion | 3/19 autoimmune_hepatitis  3/19 cardiac_failure_congestive  3/19 cardiomyopathy  3/19 embolism  3/19 hypoxia  3/19 left_ventricular_dysfunction  3/19 pleural_effusion  3/19 troponin_increased  4/19 diarrhoea  5/19 atrial_fibrillation | 6/19 |
|  | Pembrolizumab | 22/2686 | atrial_fibrillation;benign_prostatic_hyperplasia;cardiogenic_shock;conjunctivitis;malignant_neoplasm_progression;multiple_organ_dysfunction_syndrome;myocarditis;pericardial_effusion;pneumonia;product_use_issue;sepsis;septic_encephalopathy;septic_shock  atrioventricular_block_complete;autoimmune_myocarditis  atrioventricular_block_complete;autoimmune_myocarditis;myocarditis;ventricular_arrhythmia  atrioventricular_block_complete;myocarditis;myositis  blood_creatine_phosphokinase_increased;intracardiac_thrombus;liver_disorder;myocarditis;troponin_increased  death;fatigue;myocarditis  death;myocarditis  hepatic_enzyme_abnormal;immune_mediated_myocarditis;myasthenic_syndrome;serotonin_syndrome;transaminases_increased  hepatitis;myocarditis;myositis;death  immune_mediated_adverse_reaction;myocarditis  immune_mediated_adverse_reaction;myocarditis;myositis  immune_mediated_myocarditis;immune_mediated_myositis;death  autoimmune_haemolytic_anaemia;autoimmune_myocarditis;encephalitis;myasthenia_gravis;transaminases_increased  myasthenia_gravis;myocarditis;myositis;pneumonitis;death  autoimmune_haemolytic_anaemia;autoimmune_myocarditis;encephalitis;myasthenia_gravis;transaminases_increased  myocarditis;death X 3 cases  myocarditis X 4 cases | 1/22 septic_shock  1/22 serotonin_syndrome  1/22 troponin_increased  1/22 ventricular_arrhythmia  1/22 autoimmune_haemolytic_anaemia  2/22 encephalitis  3/22 atrioventricular_block_complete  3/22 myasthenia_gravis  3/22 transaminases_increased  4/22 myositis | 8/22 |
|  | Nivolumab | 34/3239 | atrioventricular_block_complete;myocarditis  autoimmune_myocarditis;autoimmune_myositis;death  autoimmune_myocarditis;blood_creatine_phosphokinase_increased;blood_creatine_phosphokinase_mb_increased;colitis;myocardial_infarction;myositis;pneumonitis;respiratory_failure;troponin_increased;death  autoimmune_myocarditis;malignant_neoplasm_progression;meningitis;pneumonitis;death  autoimmune_myocarditis;pharyngeal_haemorrhage;syncope;vomiting;death  blood_creatine_phosphokinase_increased;blood_lactate_dehydrogenase_increased;coronary_artery_stenosis;fatigue;hypertransaminasaemia;muscular_weakness;myalgia;myocarditis;myositis;troponin_increased;death  blood_pressure_decreased;hepatic_function_abnormal;myocarditis;death  chest_pain;myocarditis;pneumonitis;skin_lesion  c_reactive_protein_increased;chest_pain;dyspnoea;fibrin_d_dimer_increased;malignant_melanoma;myocarditis;n_terminal_prohormone_brain_natriuretic_peptide_increased;troponin_t_increased  diaphragm_muscle_weakness;myocarditis;myositis;respiratory_failure  duodenitis;hepatitis;myasthenia_gravis;myocarditis  eosinophilic_myocarditis  hepatic_enzyme_increased;hypoxia;myocarditis;myositis  hypertransaminasaemia;myocarditis;toxic_neuropathy;death  immune_mediated_myocarditis;muscular_weakness  mechanical_ventilation;myasthenia_gravis;myocarditis;myositis;rhabdomyolysis  muscle_necrosis;muscular_weakness;myasthenia_gravis;myocarditis  muscular_weakness;myasthenia_gravis;myocarditis;myositis;transfusion  myasthenia_gravis_crisis;myocarditis;death  myasthenia_gravis;myocarditis  myasthenic_syndrome;myocarditis;myositis;death  myocardial_infarction;myocarditis  drug_eruption;myasthenia_gravis;myocarditis;myositis X 2 cases  myocarditis;myositis X 2 cases  myocarditis;right_ventricular_failureX 2 cases  myocarditis X 6 cases | 2/34 blood_creatine_phosphokinase_increased  2/34 hypertransaminasaemia  2/34 myocardial_infarction  2/34 respiratory_failure  2/34 right_ventricular_failure  2/34 troponin_increased  3/34 pneumonitis  4/34 muscular_weakness  7/34 myasthenia_gravis  11/34 myositis | 9/34 |
|  | Ipilimumab+Nivolumab | 58/3493 | acute_kidney_injury;autoimmune_myocarditis;cardiogenic_shock;hypotension;rash_generalised  alanine_aminotransferase_increased;aspartate_aminotransferase_increased;atrioventricular_block_complete;bundle_branch_block_right;cardiac_arrest;diplopia;dyspnoea;ejection_fraction_decreased;electrocardiogram_qrs_complex_prolonged;eyelid_ptosis;facial_paralysis;fatigue;gaze_palsy;heart_alternation;myasthenia_gravis;myocarditis;orthostatic_hypotension;presyncope;respiratory_fatigue;rhabdomyolysis;troponin_increased;ventricular_tachycardia;vision_blurred;death  atrioventricular_block;cardiac_arrest;cardiac_pacemaker_insertion;hypoxia;myocarditis;myositis;death  autoimmune_colitis;autoimmune_myocarditis;chest_pain;chromaturia;dyspnoea;pneumonitis;rash_generalised;respiratory_failure;trifascicular_block;death  autoimmune_colitis;lymphocytic_hypophysitis;myocarditis  autoimmune_myocarditis  autoimmune_myocarditis;left_ventricular_dysfunction  autoimmune_thyroiditis;hypophysitis;myocarditis;therapeutic_response_decreased  blood_prolactin_abnormal;chest_pain;covid_19;dyspepsia;encephalitis;fatigue;headache;hypophysitis;metastases_to_liver;myocarditis;palpitations;rash;uveitis  death;muscular_weakness;myocarditis;neck_pain  death;myocarditis  diffuse_alveolar_damage;encephalitis;granuloma;liver_disorder;lymphocytosis;malignant_neoplasm_progression;myocarditis;pneumonitis;sarcoidosis;death  dry_eye;myocarditis;death  endocarditis;myocardial_necrosis_marker_increased;myocarditis;ventricular_arrhythmia;death  extraocular_muscle_paresis;myocarditis;pericardial_effusion;pleural_effusion;small_intestinal_haemorrhage;death  goitre;myocarditis;thyroiditis_acute  hepatocellular_injury;hyperthyroidism;hypophysitis;myocarditis;troponin_increased  hyperthyroidism;hypothyroidism;myocarditis;myositis  hyperthyroidism;myocarditis;myositis  malignant_neoplasm_progression;myocarditis;transaminases_increased  muscular_weakness;myocarditis;myositis;death  myasthenia_gravis;myocarditis;myositis;transaminases_increased  myocardial_infarction;myocarditis;death  myocarditis;myositis;renal_failure;rhabdomyolysis;death  myocarditis;thyroiditis  arthralgia;fatigue;myocarditis;myositis;vertigo;visual_impairment  autoimmune_hepatitis;myocarditis;myositis;rash_maculo_papular;thrombocytopenia;death 2 cases  autoimmune_myocarditis;death x 2 cases  hypophysitis;myocarditis;neuritis;sudden_cardiac_death;transaminases_increased x 2 cases  metastases_to_central_nervous_system;myocarditis;death x 2 cases  myocarditis;myositis;death x 2 cases  myocarditis;ventricular_tachycardia x 2 cases  myocarditis;myositis X 3 cases  myocarditis X 5 cases  myocarditis;death 11 cases | 2/58 sudden_cardiac_death  2/58 thrombocytopenia  2/58 troponin_increased  2/58 visual_impairment  3/58 hyperthyroidism  3/58 ventricular_tachycardia  4/58 fatigue  4/58 transaminases_increased  5/58 hypophysitis  15/58 myositis | 31/58 |
| NSCLC | Nivolumab | 87/9432 | acute_respiratory_failure;autoimmune_myocarditis;autoimmune_myositis;cardiogenic_shock;hepatocellular_injury;muscular_weakness;death  anaemia;atrioventricular_block;bradycardia;cardiac_pacemaker_insertion;death;eyelid_ptosis;gastrointestinal_haemorrhage;hepatic_function_abnormal;muscular_weakness;myocarditis;optic_neuritis;rash;therapeutic_embolisation  aortic_stenosis;cardiac_failure;dyspnoea;hyperpyrexia;lichen_planus;malignant_neoplasm_progression;myocarditis;pericardial_effusion;pleural_effusion;pulmonary_hypertension  arrhythmia;infarction;myocarditis;death  arteriosclerosis_coronary_artery;coronary_artery_disease;myocarditis;oesophageal_candidiasis;vascular_stent_thrombosis  asthenia;atrial_fibrillation;death;myocarditis;myopathy;myositis;pain  atrial_fibrillation;bradycardia;bundle_branch_block_left;bundle_branch_block_right;cerebral_ischaemia;dizziness;dyspnoea_exertional;fall;malignant_neoplasm_progression;myocarditis;pericardial_effusion;pericarditis;sinus_node_dysfunction;sinus_tachycardia  atrial_fibrillation;bradycardia;bundle_branch_block_left;bundle_branch_block_right;cerebral_ischaemia;malignant_neoplasm_progression;myocarditis;pericardial_effusion;sinus_node_dysfunction;sinus_tachycardia  atrial_fibrillation;myocarditis;optic_neuritis  atrioventricular_block_complete;cardiac_failure;hypothyroidism;myocarditis  atrioventricular_block_complete;myocarditis  atrioventricular_block;muscular_weakness;myocarditis  autoimmune_disorder;autoimmune_myocarditis;urinary_tract_infection;death  autoimmune_myocarditis;cardiac_failure_acute;death  autoimmune_myocarditis;cardiac_failure_congestive;death  autoimmune_myocarditis;conduction_disorder;malignant_neoplasm_progression;pneumonitis;transaminases_increased;death  autoimmune_thyroiditis;diabetic_ketoacidosis;hyperthyroidism;myocarditis  autonomic_nervous_system_imbalance;cardiac_failure_acute;hypotension;myocarditis;nervous_system_disorder  blood_creatine_phosphokinase_increased;cardiac_failure;electrocardiogram_abnormal;myalgia;myocarditis;myositis;troponin_increased  bronchial_disorder;inflammation;myocardial_infarction;myocarditis;pulmonary_embolism;septic_shock;staphylococcal_infection;stress_cardiomyopathy  cardiac_failure_congestive;malignant_neoplasm_progression;myocarditis;pulmonary_embolism;thrombocytopenia;troponin_i_increased;troponin_t_increased;death  cardiac_failure;death;myocarditis  cardiac_pacemaker_insertion;myasthenia_gravis;myocarditis  cardio_respiratory_arrest;hepatocellular_injury;myocarditis;prescribed_underdose;rhabdomyolysis;death  chest_pain;dyspnoea;myocarditis;n_terminal_prohormone_brain_natriuretic_peptide_increased;troponin_increased  chest_pain;electrocardiogram_st_segment_elevation;myocarditis;troponin_increased  cold_agglutinins;haemolysis;myocarditis;pyrexia;rash;rheumatoid_arthritis;thrombocytopenia  colitis_ulcerative;cytomegalovirus_infection;interstitial_lung_disease;lymphopenia;myocarditis;pulmonary_tuberculosis;radiation_pneumonitis  coronary_artery_disease;myocarditis  c_reactive_protein_increased;chest_pain;dyspnoea;fibrin_d_dimer_increased;malignant_melanoma;myocarditis;n_terminal_prohormone_brain_natriuretic_peptide_increased;troponin_t_increased  cytomegalovirus_infection;interstitial_lung_disease;lymphopenia;myocarditis;pulmonary_tuberculosis;radiation_pneumonitis  diplopia;dysphagia;dysphonia;eyelid_ptosis;myocarditis;myositis;ophthalmoplegia  dizziness;myocarditis;myositis  eye_disorder;malaise;myasthenia_gravis;myocarditis  gastrointestinal_perforation;hernia;intestinal_obstruction;intestinal_resection;myocarditis  general_physical_health_deterioration;myocarditis;death  hepatocellular_injury;muscular_weakness;myocarditis;myositis;death  malignant_neoplasm_progression;myocarditis;troponin_t_increased  muscular_weakness;myocarditis;myositis;renal_failure  myocarditis;myositis  myocarditis;pneumonitis  myocarditis;pulmonary_vasculitis  myocarditis;sudden_death  bronchitis;cardiac_failure;hyperthyroidism;myocarditis;pleural_effusion x 2 cases  cardiac_failure_acute;chest_pain;myocardial_infarction;myocarditis x 2 cases  cardiac_failure_congestive;myocarditis;thyroiditis x 2 cases  colitis_ulcerative;cytomegalovirus_infection;lymphopenia;myocarditis;pulmonary_tuberculosis;radiation_pneumonitis x 2 cases  cytomegalovirus_infection;interstitial_lung_disease;lymphopenia;myocarditis;pulmonary_tuberculosis x 2 cases  myocarditis;myopathy;death x 2 cases  myocarditis;type_1_diabetes_mellitus;death x 2 cases  myocarditis;myositis;death x 3 cases  myocarditis;pericarditis;pericarditis_constrictive x 3 cases  myasthenia_gravis;myocarditis x 4 cases  myocarditis x 10 cases  myocarditis;death x 10 cases | 4/87 pericarditis  4/87 radiation_pneumonitis  5/87 chest_pain  5/87 muscular_weakness  5/87 cardiac_failure  5/87 cytomegalovirus_infection  5/87 lymphopenia  6/87 myasthenia_gravis  6/87 pulmonary_tuberculosis  10/87 myositis | 30/87 |
|  | Nivolumab + ipilimumab | 3/511 | arrhythmia;autoimmune_myocarditis;cardiogenic_shock;electrolyte_imbalance;intentional_product_use_issue;multiple_organ_dysfunction_syndrome;myositis;rhabdomyolysis;death  atrioventricular_block_complete;blood_creatine_phosphokinase_increased;dyspnoea;hypothyroidism;liver_disorder;malaise;myocarditis;rash;renal_disorder  interstitial_lung_disease;myasthenia_gravis;myocarditis;off_label_use | 1/3 intentional_product_use_issue  1/3 interstitial_lung_disease  1/3 liver_disorder  1/3 malaise  1/3 multiple_organ_dysfunction_syndrome  1/3 myasthenia_gravis  1/3 rash  1/3 renal_disorder  1/3 rhabdomyolysis  1/3 myositis | 1/3 |
|  | Nivolumab + platinum doublet | 3/155 | colitis;myocarditis;death  myocarditis;death x 2 cases | 1/3 colitis | 3/3 |
|  | Atezolizumab | 16/1098 | acute_myocardial_infarction;cardiac_arrest;cardiac_failure;cardiogenic_shock;myocarditis;ventricular_fibrillation  aortitis;chest_pain;myocarditis  blood_creatine_phosphokinase_increased;myocarditis  cardio_respiratory_arrest;myocarditis;rhabdomyolysis;death  cerebrovascular_accident;muscular_weakness;myocarditis;neurological_decompensation;death  cerebrovascular_accident;myocarditis  cholestasis;death;liver_disorder;myocarditis;pneumonia  cholestasis;liver_disorder;myocarditis;pneumonia  hepatotoxicity;myocarditis;death  myocarditis;death  myocarditis;troponin_increased  arthralgia;myocarditis;pyrexia;death x2 cases  cardiac_failure;hypothyroidism;myocarditis;death x 3 cases | 1/16 troponin_increased  1/16 ventricular_fibrillation  2/16 arthralgia  2/16 cerebrovascular_accident  2/16 cholestasis  2/16 liver_disorder  2/16 pneumonia  2/16 pyrexia  3/16 hypothyroidism  4/16 cardiac_failure | 10/16 |
|  | Atezolizumab+carboplatin+paclitaxel | 3/207 | haemoptysis;myocarditis  myocarditis  myocarditis;death | 1/3 haemoptysis | 1/3 |
|  | Durvalumab | 5/1278 | autoimmune_hepatitis;myocarditis;death  bundle_branch_block_left;cardiac_failure_acute;myocarditis  hepatic_function_abnormal;muscular_weakness;myocarditis  myocarditis  myocarditis;necrotising_myositis | 1/5 autoimmune_hepatitis  1/5 bundle_branch_block_left  1/5 cardiac_failure_acute  1/5 hepatic_function_abnormal  1/5 muscular_weakness | 1/5 |
|  | Pembrolizumab+carboplatin+paclitaxel | 4/616 | cardiac_failure;myocarditis;shock;silent_myocardial_infarction  decreased_appetite;depression;liver_disorder;myasthenia_gravis;myocarditis;myositis  cardiac_failure;myocarditis;shock;silent_myocardial_infarction x2 cases | 1/4 adverse_event  1/4 decreased_appetite  1/4 depression  1/4 liver_disorder  1/4 myasthenia_gravis  1/4 myositis  3/4 cardiac_failure  3/4 shock  3/4 silent_myocardial_infarction | 0/4 |
|  | Pembrolizumab + pemetrexed + carboplatin or cisplatin | 22/892 | autoimmune_myocarditis;cardiac_failure;cardiac_failure_chronic;congestive_cardiomyopathy;drug_intolerance;inappropriate_schedule_of_product_administration;left_ventricular_dysfunction;lymphadenopathy_mediastinal;off_label_use;performance_status_decreased;pleural_effusion;ventricular_tachycardia  cardiac_arrest;coronary_vascular_graft_occlusion;dehydration;diarrhoea;ejection_fraction_decreased;myocarditis;pleural_effusion;stress_cardiomyopathy;supraventricular_tachycardia;death  hypophysitis;hypothyroidism;immune_mediated_myocarditis;myositis;nausea;nephritis;rash;renal_impairment  hypophysitis;hypothyroidism;myocarditis;myositis;nephritis;rash;renal_impairment  hypophysitis;hypothyroidism;myocarditis;myositis;rash;renal_impairment  myocarditis  myocarditis;myositis;respiratory_failure;death    immune_mediated_myocarditis;death 2x cases  autoimmune_myocarditis;cardiac_failure;cardiac_failure_chronic;congestive_cardiomyopathy;drug_intolerance;inappropriate_schedule_of_product_administration;left_ventricular_dysfunction;lymphadenopathy_mediastinal;performance_status_decreased;pleural_effusion;ventricular_tachycardia 3x cases    myocarditis;death 3x cases  myocarditis;myositis;product_use_in_unapproved_indication;respiratory_failure;death x3 cases  cardiogenic_shock;myocarditis 4x cases | 1/22 cardiac_arrest  1/22 coronary_vascular_graft_occlusion  1/22 dehydration  1/22 diarrhoea  1/22 ejection_fraction_decreased  1/22 nausea  1/22 off_label_use  1/22 stress_cardiomyopathy  1/22 supraventricular_tachycardia  2/22 nephritis  3/22 hypophysitis  3/22 hypothyroidism  3/22 immune_mediated_myocarditis  3/22 product_use_in_unapproved_indication  3/22 rash  3/22 renal_impairment  4/22 autoimmune_myocarditis  4/22 cardiac_failure  4/22 cardiac_failure_chronic  4/22 cardiogenic_shock  4/22 congestive_cardiomyopathy  4/22 drug_intolerance  4/22 inappropriate_schedule_of_product_administration  4/22 left_ventricular_dysfunction  4/22 lymphadenopathy_mediastinal  4/22 performance_status_decreased  4/22 respiratory_failure  4/22 ventricular_tachycardia  5/22 pleural_effusion  7/22 myositis | 10/22 |
| RCC | Pembrolizumab+axitinib | 2/163 | atrioventricular_block_complete;coronary_artery_stenosis;myocarditis  dependence_on_respirator;hepatitis;myelitis;myocarditis | 1/2 atrioventricular_block_complete  1/2 coronary_artery_stenosis  1/2 dependence_on_respirator  1/2 hepatitis  1/2 myelitis | 0/2 |
|  | Avelumab+axitibib | 1/52 | myocarditis;death |  | 1/1 |
|  | Sunitinib | 1/13624 | cardiomyopathy;fatigue;myocarditis;shock;death | 1/1 cardiomyopathy  1/1 fatigue  1/1 shock | 1/1 |

Table S3. Co-occurring AEs, and death by any cause in myocarditis cases associated with ICI use in various types of cancer.

Table S4

|  | **ICI** | **Death in myocarditis cases, n** | **Myocarditis cases, n** | **Death by any cause in myocarditis cases (%)** | **Death in all cases, n** | **Death by any cause all cases (%)** |
| --- | --- | --- | --- | --- | --- | --- |
| **Melanoma** | Ipilimumab | 6 | 19 | 31.6 | 857 | 18.4 |
|  | Pembrolizumab | 8 | 22 | 36.4 | 580 | 21.6 |
|  | Nivolumab | 9 | 34 | 26.5 | 781 | 24.1 |
|  | Ipilimumab+Nivolumab | 31 | 58 | 53.4 | 695 | 19.9 |
|  |  |  |  |  |  |  |
| **NSCLC** | Nivolumab | 30 | 87 | 34.5 | 2811 | 29.8 |
|  | Ipilimumab+nivolumab | 1 | 3 | 33.3 | 161 | 31.5 |
|  | Nivolumab+platinum doublet | 3 | 3 | 100.0 | 49 | 31.6 |
|  | Atezolizumab | 10 | 16 | 62.5 | 349 | 31.8 |
|  | Atezolizumab+carboplatin+paclitaxel+bevacizumab | 1 | 3 | 33.3 | 67 | 32.4 |
|  | Durvalumab | 1 | 5 | 20.0 | 298 | 23.2 |
|  | Pembrolizumab+carboplatin+paclitaxel | 0 | 4 | 0.0 | 184 | 29.8 |
|  | Pembrolizumab + pemetrexed + carboplatin or cisplatin | 10 | 22 | 45.5 | 257 | 28.8 |
|  |  |  |  |  |  |  |
| **RCC** | Pembrolizumab+axitinib | 0 | 2 | 0.0 | 14 | 8.6 |
|  | Avelumab+axitibib | 1 | 1 | 100.0 | 8 | 15.4 |
|  |  |  |  |  |  |  |
|  | All ICI reports | 101 | 253 | 39.9 | 7111 | 25.0 |

Table S4. Occurrence of death by any cause in FAERS/AERS myocarditis reports in ICI patients compared to all ICI AE cases.

Table S5

| **Adverse event** | **% occurrence in myocarditis patients** |
| --- | --- |
| Myositis | 17.6 |
| Myasthenia_gravis | 8.2 |
| cardiac_failure (chronic, acute, congestive) | 10.4 |
| Pneumonitis | 4.7 |
| troponin_increased | 4.3 |

Table S5. Top five most frequent adverse events reported to co-occur with myocarditis.

Table S6

|  | Tofacitinib | Sunitinib | Axitinib | Imatinib | Ruxolitinib | Erlotinib | Nilotinib | Ibrutinib | Dasatinib | Lapatinib |
| --- | --- | --- | --- | --- | --- | --- | --- | --- | --- | --- |
| Total n | 73602 | 25,196 | 6,683 | 38,014 | 20,585 | 25,906 | 15930 | 21,218 | 17026 | 4895 |
| Myocarditis reports n,(%) | 7 (0.01) | 5 (0.02) | 2 (0.03) | 7 (0.02) | 0 (0.00) | 0 (0.00) | 2 (0.01) | 7 (0.03) | 5 (0.03) | 0 (0.00) |

Table S6. FAERS/AERS reported myocarditis cases in 10 most frequently reported kinase inhibitor monotherapy cases.
